# A neutrophil/TGF-**β** axis limits the pathogenicity of allergen-specific CD4^+^ T cells

**DOI:** 10.1172/jci.insight.150251

**Published:** 2022-02-22

**Authors:** Gregory S. Whitehead, Seddon Y. Thomas, Keiko Nakano, Derek J. Royer, Catherine G. Burke, Hideki Nakano, Donald N. Cook

**Affiliations:** Immunity, Inflammation, and Disease Laboratory, National Institute of Environmental Health Sciences (NIEHS), National Institutes of Health (NIH), Research Triangle Park, North Carolina, USA.

**Keywords:** Immunology, Inflammation, Asthma, Neutrophils, Th2 response

## Abstract

The intensity and longevity of inflammatory responses to inhaled allergens is determined largely by the balance between effector and regulatory immune responses, but the mechanisms that determine the relative magnitudes of these opposing forces remain poorly understood. We have found that the type of adjuvant used during allergic sensitization has a profound effect on both the nature and longevity of the pulmonary inflammation triggered by subsequent reexposure to that same provoking allergen. TLR ligand adjuvants and house dust extracts primed immune responses characterized by a mixed neutrophilic and eosinophilic inflammation that was suppressed by multiple daily allergen challenges. During TLR ligand–mediated allergic sensitization, mice displayed transient airway neutrophilia, which triggered the release of TGF-β into the airway. This neutrophil-dependent production of TGF-β during sensitization had a delayed, suppressive effect on eosinophilic responses to subsequent allergen challenge. Neutrophil depletion during sensitization did not affect numbers of Foxp3^+^ Tregs but increased proportions of Gata3^+^CD4^+^ T cells, which, upon their transfer to recipient mice, triggered stronger eosinophilic inflammation. Thus, a neutrophil/TGF-β axis acts during TLR-mediated allergic sensitization to fine-tune the phenotype of developing allergen-specific CD4^+^ T cells and limit their pathogenicity, suggesting a novel immunotherapeutic approach to control eosinophilia in asthma.

## Introduction

Allergic asthma is a widespread, chronic inflammatory disease of the airways characterized by reversible airway obstruction, airway hyperresponsiveness (AHR), and inflammation (1). Approximately 8% of the adult US population currently has asthma, and it is estimated that globally, more than 300 million people have this disease, exacting an enormous burden on economies and quality of life worldwide (reviewed in ref. 2). The prevalence of asthma has been steadily increasing over the last several decades, suggesting that a changing environment is driving this increase. Indeed, many types of environmental exposures have been associated with an increased incidence of asthma, but in most cases, the mechanistic links between these exposures and asthma pathogenesis are poorly understood. This is in part because asthma is not a single disease, but rather a spectrum of disease pathologies that can be distinguished by multiple parameters that include the nature of airway inflammation. Many patients display eosinophilic inflammation associated with type 2 immune responses, but almost half of patients display a noneosinophilic form of asthma, often with predominant neutrophilia (3, 4). It is likely that actions of allergen-specific T helper 2 (Th2) and Th17 cells play a major role in driving eosinophilic and neutrophilic inflammation, respectively. The concept of asthma “endotypes” further postulates that different forms of asthma arise from perturbations of distinct cellular and molecular pathways (5, 6). A better understanding of how specific pathways give rise to distinct asthma endotypes should in turn lead to novel targets and more precise strategies for therapeutic intervention.

The development of asthma can be regarded as having 2 phases. The first is an asymptomatic phase during which inhaled allergens are taken up by lung dendritic cells, which then migrate to regional lymph nodes (LNs), where they present allergen-derived peptides to naive T cells. T cells with cognate receptors for those peptides differentiate into effector T cells and undergo proliferation. In the second phase, reexposure to the same allergen triggers migration of those T cells to the lung, where they release cytokines and other molecules that drive airway inflammation. The microenvironment within which allergen-specific T cells develop likely affects the nature of their responses to the provoking allergen, and this is well established for cells that develop in vitro. Thus, IL-12 and IFN-γ induce Th1 cells, IL-4 drives Th2 cells, TGF-β elicits T regulatory cells (Tregs), and a combination of TGF-β with either IL-1 or IL-6 induces Th17 differentiation. However, very little is known regarding how local concentrations of these cytokines are generated in vivo and how their levels are affected by different adjuvants. Previous work has revealed that protease adjuvants promote a type 2 cytokine–driven, eosinophilic inflammation of the airway, whereas TLR ligands, such as LPS and flagellin, promote both type 2 and Th17 responses that lead to a mixed neutrophilic and eosinophilic inflammation (7). In agreement with this observation, LPS contributes to the allergenicity of house dust mite allergen (8), and household levels of bacteria and endotoxin correlate with asthma prevalence (9). Paradoxically, however, several studies have shown that exposure to endotoxin, especially during early life in agricultural settings, is associated with protection from developing asthma in later life. This is consistent with previous observations that inhalation of LPS can induce Treg proliferation (10) and suppress asthma-like responses in mice (11). An improved understanding of the cellular and molecular mechanisms that underlie the ability of LPS to both promote allergic sensitization and protect against the development of asthma might lead to novel therapeutic strategies to prevent or mitigate this disease.

In the present report, we show that allergic airway inflammation was suppressed by prolonged allergen challenges in mice sensitized to OVA using LPS as an adjuvant but not in mice sensitized using a protease as an adjuvant. This suppression was dependent on acute airway neutrophilia and consequent TGF-β release into the airway that occurred during the initial LPS-mediated sensitization phase. Neutrophil depletion during allergic sensitization blocked TGF-β production and prevented the suppression of allergic responses to prolonged allergen challenge. Further, recombinant mouse TGF-β delivered to the airways during sensitization restored the immunosuppression that had been prevented by neutrophil depletion. Analysis of CD4^+^ T cells revealed that the presence of neutrophils in the airway during allergic sensitization did not affect the development or proliferation of the Th2 cells during the first encounters with the allergen, but neutrophils had a long-lasting impact on suppressing the expansion and activity of Gata3^+^ Th2 cells. Thus, neutrophil influx into the airway and consequent production of TGF-β during allergic sensitization blunted the pathogenicity of Th2 cells and limited the longevity of asthma.

## Results

### Inhalation of LPS triggers both effector and regulatory responses to inhaled allergens.

**Figure 1:**
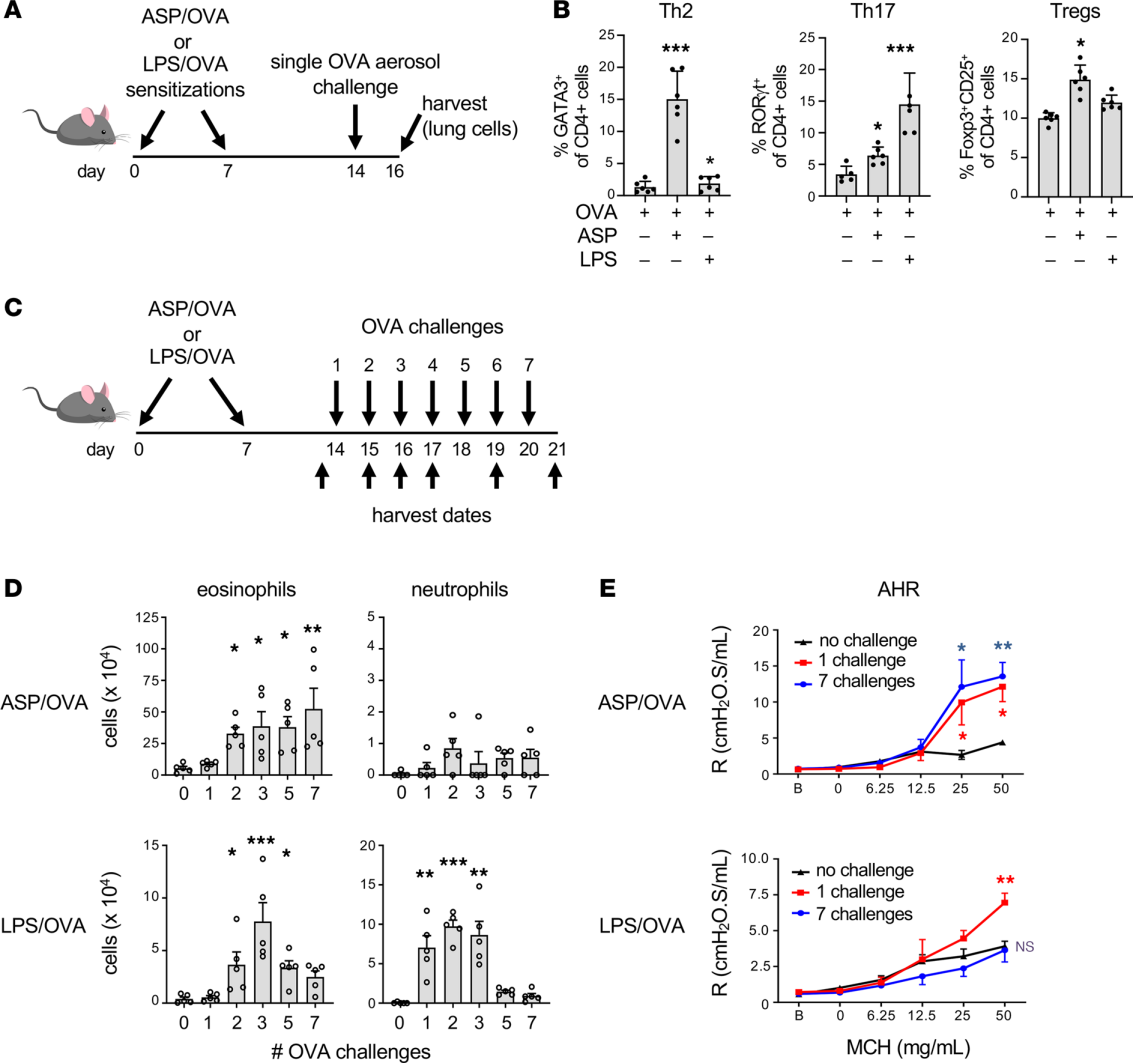
The nature and longevity of allergic responses depend on the type of adjuvant used during sensitization. (**A** and **B**) Mice were sensitized to OVA using either *Aspergillus oryzae* (ASP) or LPS as the adjuvant, then challenged on a single occasion with aerosolized OVA. (**A**) Timeline of allergic sensitizations, single challenge, and harvest. (**B**) Percentages of Th2 (Gata3^+^) cells, Th17 (RORγt^+^) cells, and Tregs (Foxp3^+^CD25^+^) in CD4^+^ T cells from lungs of mice 2 days after a single challenge. (*n* = 6 mice/group). (**C**) Timeline of sensitizations, daily challenges, and harvest. (**D**) Airway inflammation 1 day after the last of the indicated number of OVA challenges (*n* = 5 mice/group). (**E**) Airway hyperresponsiveness (AHR) measured 2 days after a single challenge or 1 day after the last of 7 daily challenges (*n* = 6 mice/group). Data shown represent mean values ± SEM from a single experiment, representative of 2, except for **B**, a single experiment. *P < 0.05; **P < 0.01; ***P < 0.001; unchallenged (0) group vs. allergen-challenged groups; Kruskal-Wallis 1-way ANOVA with Dunn’s multiple-comparison test. R, resistance; MCH, methacholine.

Previous studies have shown that mice sensitized to an allergen using protease adjuvants develop a predominantly eosinophilic inflammation of the airway upon subsequent allergen challenge, whereas mice sensitized using the TLR ligands, LPS or flagellin, develop a mixed eosinophilic and neutrophilic inflammation after a single challenge (7). This suggests that as adjuvants, proteases and LPS prime predominantly Th2 and Th17 responses, respectively. To test this, we performed intracellular staining of CD4^+^ T cells for Th lineage-specific transcription factors following allergic sensitization and a single OVA challenge (Figure 1A). Mice previously sensitized to OVA using a mix of proteases from *Aspergillus oryzae* (ASP/OVA) as the adjuvant had very high percentages of Th2 cells after challenge, based on intracellular staining of Gata3, and had relatively low percentages of retinoic acid receptor–related orphan receptor γt–positive (RORγt^+^) Th17 cells (Figure 1B and Supplemental Figure 1; supplemental material available online with this article; https://doi.org/10.1172/jci.insight.150251DS1). Conversely, mice sensitized using LPS from *E*. *coli* (LPS/OVA) as the adjuvant had low percentages of Th2 cells and a high proportion of Th17 cells. Of note, no adjuvant-specific differences were seen in the percentages of Foxp3^+^ Tregs.

We next investigated whether different classes of adjuvants also affect the longevity of immune responses to inhaled allergens. Mice were again sensitized through the airways to OVA using ASP/OVA or LPS/OVA, then subjected to various numbers of daily challenges with aerosolized OVA (Figure 1C). As expected, eosinophilia was higher in ASP/OVA-sensitized mice than in LPS/OVA-sensitized animals, but in both groups of mice, the number of eosinophils in the airway progressively increased with the number of allergen challenges, up to 3 challenges (Figure 1D). However, whereas additional challenges of ASP/OVA-sensitized mice further increased numbers of eosinophils in the airway, these cells declined markedly in LPS/OVA-sensitized animals after 3 challenges. A similar decline of neutrophilic inflammation was also observed in the LPS/OVA-sensitized mice that were challenged on more than 3 occasions, whereas the relatively low number of neutrophils in ASP/OVA-sensitized mice remained unchanged. As AHR is a salient feature of allergic asthma, we also studied how different types of adjuvants affect the longevity of this physiologic response to prolonged allergen challenges. Mice sensitized with ASP/OVA developed robust AHR after a single challenge, and this was sustained even after multiple daily allergen challenges (Figure 1E). By contrast, although LPS/OVA-sensitized mice developed AHR after a single challenge, it was greatly diminished after multiple allergen challenges.

**Figure 2:**
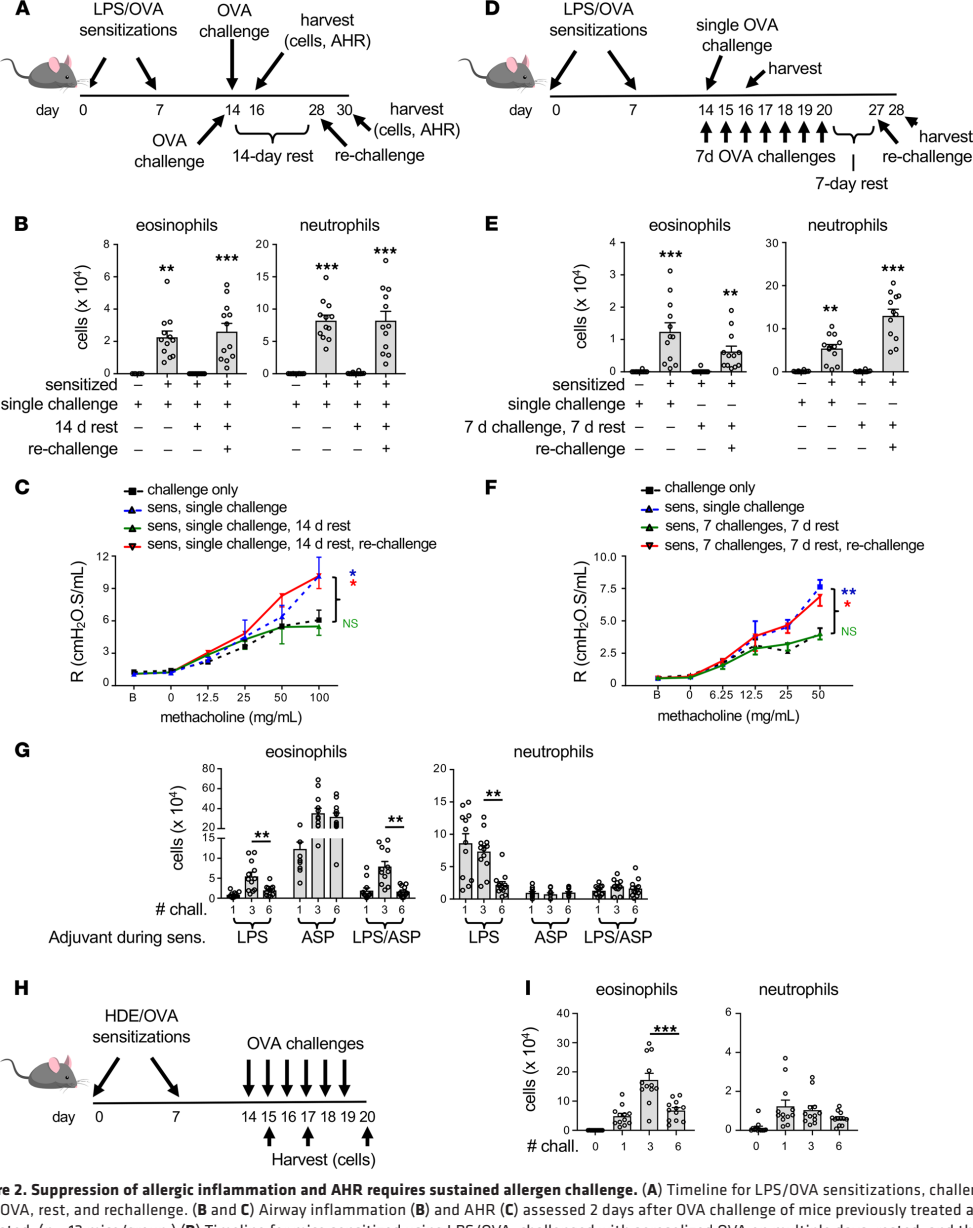
Suppression of allergic inflammation and AHR requires sustained allergen challenge. (**A**) Timeline for LPS/OVA sensitizations, challenge with OVA, rest, and rechallenge. (**B** and **C**) Airway inflammation (**B**) and AHR (**C**) assessed 2 days after OVA challenge of mice previously treated as indicated. (*n* = 12 mice/group.) (**D**) Timeline for mice sensitized using LPS/OVA, challenged with aerosolized OVA on multiple days, rested, and then rechallenged. Control mice received similar sensitizations but were challenged with OVA on a single occasion. (**E** and **F**) Airway inflammation (**E**) and AHR (**F**) 2 days after the final OVA challenge (*n* = 12 mice/group). (**G**) Cell numbers for each of the indicated leukocyte types in bronchoalveolar lavage (BAL) fluid of mice that were sensitized (sens.) using OVA together with the indicated adjuvants, LPS, ASP, or LPS/ASP, then challenged on 1, 3, or 6 occasions with OVA (*n* = 12 mice/group). (**H**) Timeline for HDE/OVA model of asthma. (**I**) Airway inflammation in mice sensitized with HDE/OVA, then challenged daily for the indicated number of days. Data shown represent mean values ± SEM for combined data of 2 experiments yielding similar results. *P < 0.05, **P < 0.01, ***P < 0.001; (**B**, **C**, **E**, and **F**) challenged only vs. sensitized + challenged mice; 1-way ANOVA with Tukey’s multiple-comparison test. (**H**) Two-way ANOVA with Tukey’s multiple-comparison test.

The declining inflammatory and physiologic responses to prolonged allergen challenge in LPS/OVA-sensitized mice might have been due to the comparatively weak Th2 response in these animals or to active suppression by prolonged exposure to the allergen. To distinguish between these possibilities, we sensitized mice to LPS/OVA and challenged them on 2 occasions, 2 weeks apart. The 2-week rest period was required for the eosinophilic inflammation from the first challenge to resolve (Figure 2A). Mice treated in this way responded as vigorously to the second OVA challenge as they did to the first challenge (Figure 2B), including their development of AHR (Figure 2C). These results suggest that continued daily challenges are required for the suppression of allergic responses. To confirm this, we performed an experiment in which mice were sensitized as before with LPS/OVA but this time challenged on 7 consecutive days, then allowed to rest for 1 week before an additional, final challenge (Figure 2D). In this experiment, the multiple challenges had already begun to reduce the eosinophilic inflammation, and the 1-week rest period was sufficient for that inflammation to resolve. As expected, control mice that were challenged on 7 consecutive days and rested for 1 week, but not rechallenged, had neither robust inflammation nor AHR (Figure 2, E and F). These animals also had very low airway levels of IL-4, IL-5, and IL-17 (Supplemental Figure 2). By contrast, animals that underwent a similar challenge and rest period, but were subsequently rechallenged, displayed multiple asthma-like features, including inflammation, AHR, and cytokine production. Eosinophils were slightly lower in the group undergoing multiple challenges before the final rechallenge, but this difference was not statistically significant. Together, the data suggest that animals retain allergen-responsive effector cells whose actions are temporarily suppressed by continued daily allergen exposures (Figure 1, D and E).

To confirm that LPS/OVA sensitization promotes allergen-specific, immunosuppressive responses, we asked whether LPS, when added to a mixture of ASP/OVA, would be dominant over ASP/OVA’s ability to prime sustained inflammatory responses to OVA challenge. Animals sensitized using this LPS/ASP/OVA mixture displayed a similar pattern of eosinophilic inflammation to that seen in mice sensitized to LPS/OVA, with numbers of these cells progressively increasing after 1 or 3 daily challenges but decreasing thereafter (Figure 2G). Thus, although LPS acts as an adjuvant to promote allergen-specific Th2 and Th17 immune responses, it also triggers an immunoregulatory response that becomes dominant in the face of repeated exposure to allergen.

Common house dust contains a mixture of environmental adjuvants, including proteases and TLR ligands, such as LPS (12, 13). To determine if this natural mix of different adjuvant types also triggers immunoregulatory responses, we employed an established model utilizing house dust extracts (HDEs) as an adjuvant for sensitizing mice to OVA (14) (Figure 2H). As seen previously for LPS/OVA-sensitized mice, HDE/OVA-sensitized animals also displayed airway inflammation for up to 3 daily OVA challenges, but these responses declined with additional daily challenges (Figure 2I). Thus, common house dust also contains agents that limit the longevity of allergic responses to inhaled allergens.

### Airway neutrophils promote regulatory responses to multiple allergen challenges.

**Figure 3:**
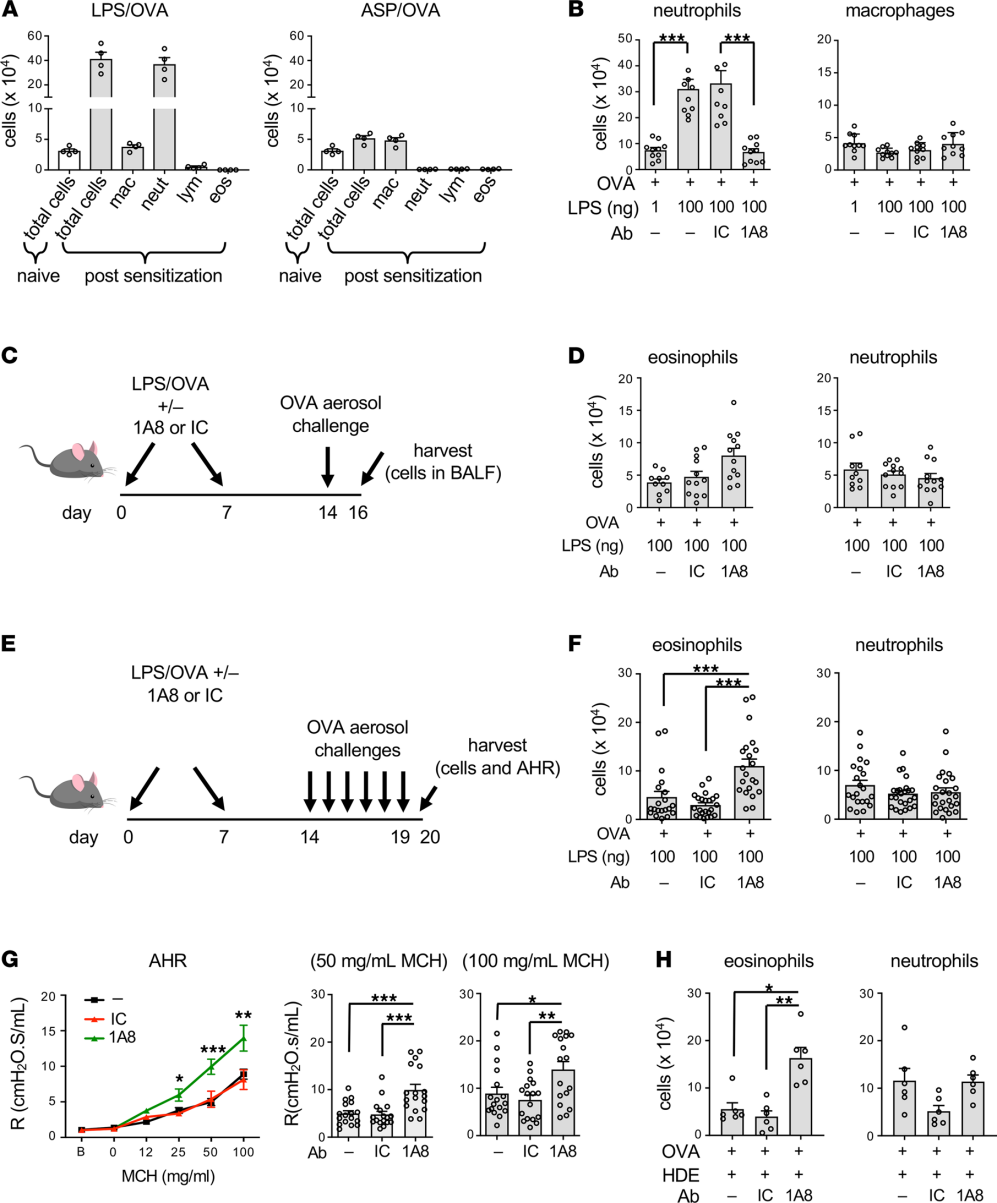
Airway neutrophilia during allergic sensitization curtails subsequent asthma-like responses to allergen challenge. (**A**) Acute inflammatory responses to inhaled LPS/OVA and ASP/OVA. Shown are mean cell numbers ± SEM for the indicated leukocytes in BAL fluid of mice 16 hours after inhalation (*n =* 4 mice/group). (**B**) Efficiency of 1A8 Ab–mediated neutrophil depletion in mice inhaling OVA together with the indicated amounts of LPS (*n* = 8 mice/group). (**A** and **B**) Data shown are from single experiments, representative of 2. (**C**) Timeline for neutrophil depletion, followed by LPS/OVA sensitizations and a single OVA challenge. (**D**) Cell numbers for the indicated leukocytes in BAL fluid 2 days after OVA challenge, as shown in **C**. Values shown represent mean ± SEM, and the data are combined from 2 experiments (*n* = 12 mice/group). (**E**–**G**) Effect of neutrophil depletion on responses to multiple OVA challenges. (**E**) Timeline for neutrophil depletion, allergic sensitization, and multiple OVA challenges. (**F**) Cell numbers for the indicated leukocytes in BAL fluid 2 days after the last of 6 OVA challenges, as shown in **E**. Values shown represent mean ± SEM, and the data are combined from 3 experiments (*n* = 18 mice/group). (**G**) Effect of neutrophil depletion prior to LPS/OVA-mediated sensitization on AHR. Airway resistance values shown represent mean ± SEM and are combined from 2 experiments (*n* = 17 mice/group). (**H**) Effect of neutrophil depletion prior to HDE/OVA sensitization. Cell numbers for the indicated leukocytes in BAL fluid of OVA-challenged mice previously sensitized using HDE/OVA as depicted in Figure 2H. Values shown represent mean ± SEM from 1 of 2 experiments yielding similar results (*n* = 6 mice/group). *P < 0.05, **P < 0.01, ***P < 0.001; 1-way ANOVA with Holm-Šídák multiple-comparison test.

In our experiments performed thus far with LPS/OVA- and ASP/OVA-sensitized mice, the different groups were challenged with aerosolized OVA in the same exposure chamber and at the same time. Therefore, the observed differences in the longevity of responses to OVA challenge must have been due to distinct cellular and molecular pathways triggered by these adjuvants at the time of allergic sensitization. Accordingly, we studied adjuvant-specific changes in the airway 16 hours postsensitization. We found that instillation of LPS/OVA into the airway caused rapid airway inflammation (Figure 3A), due almost entirely to neutrophilia. This did not occur in ASP/OVA-treated mice. These observations prompted us to investigate whether the neutrophils recruited to the airway during LPS-mediated allergic sensitization have a regulatory effect on immune responses that only becomes apparent after multiple daily allergen challenges. As expected, injection of the neutrophil-depleting antibody, anti-Ly6G (clone 1A8), significantly reduced the number of neutrophils in the airspace following LPS inhalation but did not affect macrophage number (Figure 3B). This neutrophil depletion during LPS/OVA sensitization did not significantly affect subsequent responses of mice to a single OVA challenge, although there was a trend toward increased eosinophilia (Figure 3, C and D). Antibody-mediated neutrophil depletion during sensitization did not affect numbers of neutrophils in the airway after allergen challenge. This result was not unexpected, as neutrophil numbers recover within 2 or 3 days of 1A8-mediated depletion (15). Unlike responses to a single OVA challenge, depleting neutrophils during sensitization had a dramatic effect on subsequent responses to 6 consecutive daily allergen challenges. Thus, mice in which neutrophils were depleted prior to allergic sensitization displayed a striking and selective increase in eosinophilia compared with similarly sensitized and challenged mice that received either no antibody, or the isotype control (IC) antibody, prior to sensitization (Figure 3, E and F). Similar findings were observed when we used an anti–Gr-1 antibody, which depletes both neutrophils and monocytes (Supplemental Figure 3, A and B). Neutrophil depletion during LPS/OVA sensitization also affected physiologic responses to subsequent OVA challenges, as indicated by increased AHR in these mice (Figure 3G). The ability of neutrophil depletion during allergic sensitization to increase asthma-like features during the challenge phase was not unique to the LPS/OVA model of asthma, because we also observed this in 3 additional environmentally relevant animal models, the above-described HDE/OVA model (Figure 2H and Figure 3H) and another model in which HDE served as both the adjuvant and source of allergens (Supplemental Figure 3, C and D). Likewise, instillation of bacterial flagellin into the airway together with OVA triggered transient neutrophilia (Supplemental Figure 3C), and depletion of neutrophils during flagellin-mediated allergic sensitization heightened responses to subsequent allergen challenge (Supplemental Figure 3E). Together, these observations show that airway neutrophilia during allergic sensitization has a delayed, suppressive effect on eosinophilia and AHR in multiple models of asthma.

### Neutrophil recruitment to the airway during sensitization is sufficient to limit eosinophilic responses to multiple allergen challenges.

**Figure 4:**
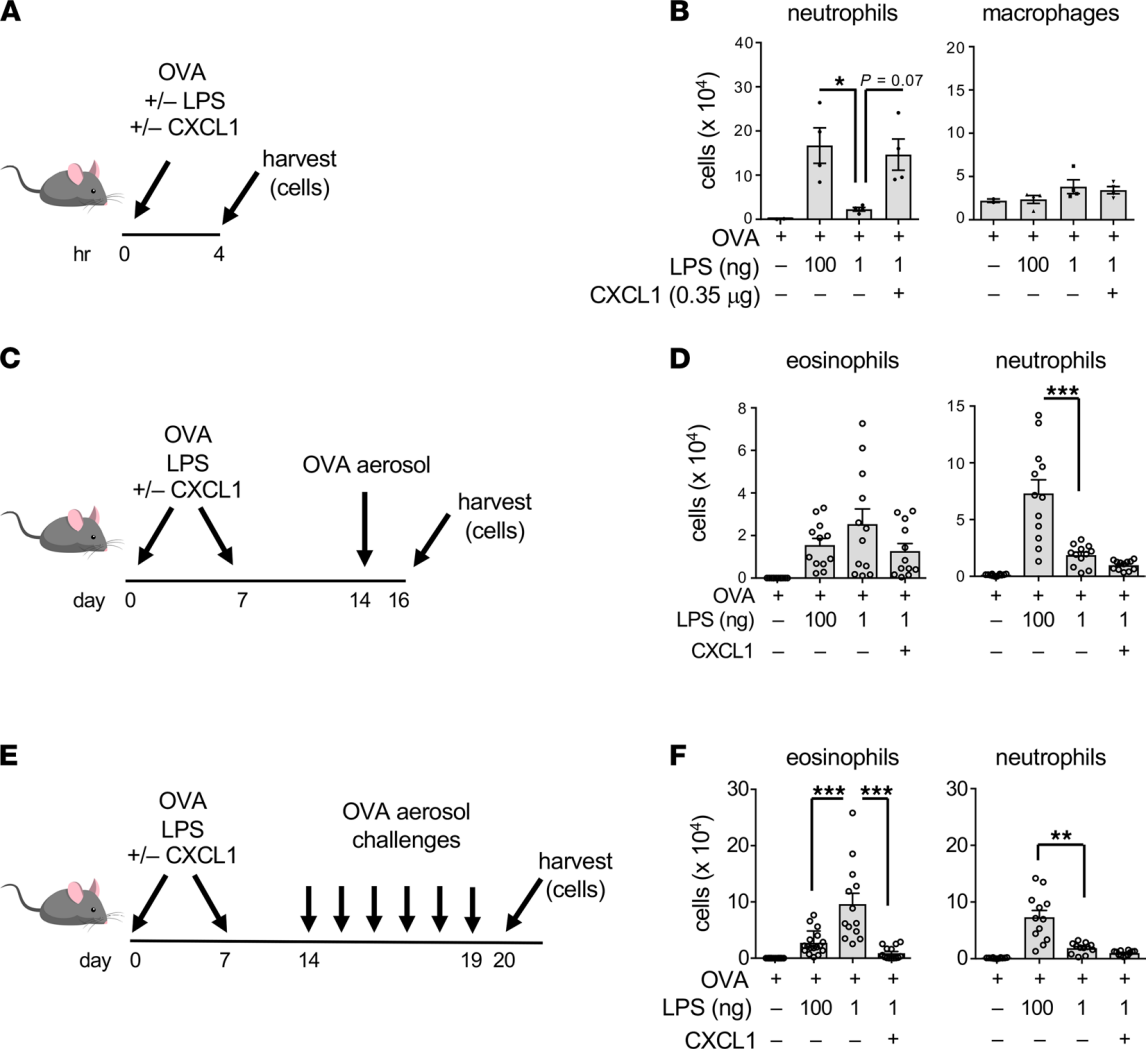
Neutrophilia during sensitization is sufficient for immunosuppression. (**A**) Timeline for sensitizations to OVA using combinations of LPS (1 and 100 ng) and CXCL1 and harvest. (**B**) Cell numbers for indicated leukocytes in the airway 4 hours after administration of the indicated reagents. Values shown represent mean ± SEM from 1 of 2 experiments yielding similar results (*n* = 4 mice/group). *P < 0.05, Kruskal-Wallis 1-way ANOVA with Dunn’s multiple-comparison test. (**C**) Timeline for neutrophil depletion, followed by allergic sensitization and a single OVA challenge. (**D**) Airway inflammation 2 days after OVA challenge of mice treated as shown in **C**. Values shown represent mean ± SEM from combined data from 2 experiments (*n* = 12 mice/group). ***P < 0.001; 1-way ANOVA with Holm-Šídák multiple-comparison test. (**E**) Timeline for multiple OVA challenges following allergic sensitization using combinations of OVA, LPS and CXCL1. (**F**) Airway inflammation 1 day after OVA challenge of mice treated as shown in **E**. Values shown represent mean ± SEM and are combined from 2 experiments. (*n* = 12 mice/group). **P < 0.01, ***P < 0.001; 1-way ANOVA with Holm-Šídák multiple-comparison test.

Having found that neutrophils recruited to the airway during sensitization are required for suppression of responses to prolonged allergen challenge, we next asked whether these cells are sufficient for that effect. Previous studies have shown that mice sensitized to OVA using very small doses of LPS (1 ng) display more sustained responses to OVA challenge than mice sensitized using higher doses (100 ng) (11). To further investigate this finding, we first confirmed that allergic sensitization using relatively low doses of LPS led to less acute airway neutrophilia than when higher doses of LPS were used (Supplemental Figure 4). We reasoned that if airway neutrophilia during sensitization is sufficient to promote immunoregulatory responses, then including a neutrophil-attracting chemokine together with very low doses of LPS during allergic sensitization might phenocopy the suppressive responses to prolonged allergen challenge seen when higher doses of LPS are used as the adjuvant. Prior to performing this experiment, we first verified that supplementation of very-low-dose LPS (1 ng) with 0.35 μg of the neutrophil-attracting chemokine, CXCL1, recruited as many neutrophils to the airway as did administration of a 100-fold higher dose of LPS (100 ng) and that there were minimal effects on macrophage numbers (Figure 4, A and B). Having established that, we sensitized mice on 2 occasions with OVA mixed with low-dose LPS and CXCL1, then challenged them on a single occasion with aerosolized OVA (Figure 4C). This inclusion of CXCL1 with low-dose LPS during allergic sensitization did not significantly reduce eosinophilic responses to a single OVA challenge, although there was a trend in that direction (Figure 4D). However, including CXCL1 during allergic sensitization did reduce the eosinophilia seen after 6 daily OVA challenges (Figure 4, E and F). Of note, inclusion of CXCL1 with small amounts of LPS during sensitization did not appear to be sufficient to drive Th17 responses, as these mice did not have significant neutrophilia after challenge. Data from this set of experiments show that airway neutrophilia during allergic sensitization is both necessary and sufficient to negatively regulate eosinophilic inflammation during prolonged allergen challenge.

### Neutrophil recruitment to the airway during allergic sensitization alters the expansion and function of CD4^+^ T cells.

**Figure 5:**
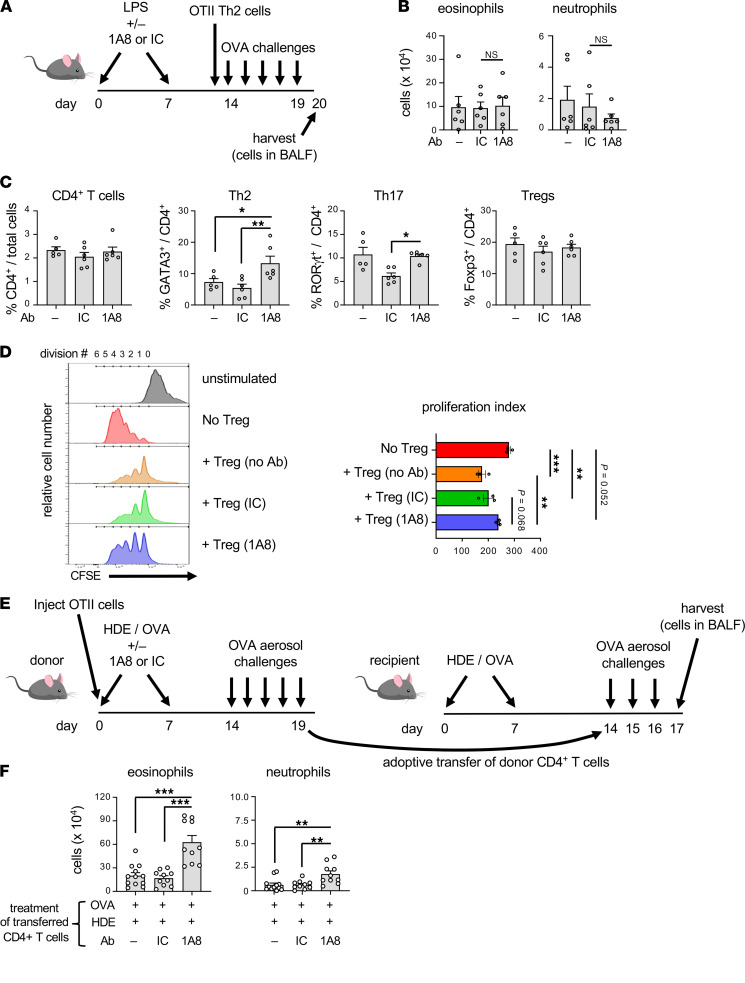
Neutrophil depletion during sensitization augments CD4^+^ T cell pathogenicity. (**A**) Timeline for neutrophil depletion, inhalation of LPS, transfer of in vitro–generated OVA-specific Th2 cells, and OVA challenges. (**B**) Cell numbers for the indicated leukocytes in BAL fluid 1 day after the final OVA challenge of mice treated as in **A** (*n* = 6 mice/group). (**C**) Effect of neutrophil depletion on cell numbers for the indicated CD4^+^ Th subsets. Shown are percentages of the indicated CD4^+^ Th cell subsets in the lungs of mice sensitized with OVA/HDE and challenged with OVA on multiple occasions, as shown in Figure 3E. (**D**) Effect of neutrophil depletion on Treg functional activity. Relative cell numbers corresponding to each generation of cell division are shown in representative histograms (left). Also shown are compiled data representing proliferative indices (right) as described in Methods. (**E**) Timeline for adoptive transfer of OT-II CD4^+^ T cells into primary recipients, their OVA sensitization and challenge, harvest of donor CD4^+^ T cells, and their transfer into secondary recipients. (**F**) Cell numbers for the indicated leukocytes in BAL fluid 1 day following the last challenge of mice treated as shown in **E** (right). Values shown represent mean ± SEM from 1 of 2 experiments yielding similar results. *P < 0.05, **P < 0.01, ***P < 0.001; Kruskal-Wallis 1-way ANOVA with Dunn’s multiple-comparison test.

In addition to their well-described roles in the initiation of immune responses, antigen-presenting cells (APCs) are also required during the challenge phase of mouse models of asthma (16). Further, the actions of APCs can be suppressed by prior exposure to a strong initial stimulus (17). We therefore considered the possibility that neutrophil recruitment to the airway promotes allergen-dependent immunosuppression by effecting APC function. To test this, we again depleted neutrophils prior to LPS inhalation using the 1A8 antibody, but in this case omitted OVA, which would have stimulated OVA-specific T cells. The impact of neutrophil depletion on APCs — as well as other cells not specific to OVA — was assessed by adoptive transfer of in vitro–generated, OVA-specific Th2 cells to the LPS-treated mice, followed by 5 consecutive daily OVA challenges (Figure 5A). No difference in eosinophilic inflammation was seen between recipients that had undergone prior 1A8-mediated neutrophil depletion and those that had not (Figure 5B). This suggests that any effects of neutrophils on nonlymphocyte, lung resident cells, such as epithelial cells or dendritic cells, are not responsible for the immunosuppression seen following prolonged allergen challenges.

We next tested whether neutrophilia during allergic sensitization affects CD4^+^ T cell pathogenicity. Mice were sensitized with HDE/OVA, with or without neutrophil depletion, then challenged daily with OVA for 5 days, and intracellular was staining performed on T cells for lineage-specific transcription factors. Although neutrophil depletion did not affect percentages of total CD4^+^ T cells in the lung, mice in which neutrophils had been depleted during allergic sensitization had increased percentages of Gata3^+^ Th2 cells compared with mice that received no antibody or IC antibody (Figure 5C and Supplemental Figure 5). Despite this increase in Gata3^+^ Th2 cells, we did not observe corresponding increases in *Il5* expression or in IL-5 protein in BAL fluid (GS Whitehead, unpublished data). It is possible that differences might have been present in particular anatomic niches or at specific times not tested. Nonetheless, the observed increase in Gata3^+^ Th2 cells is consistent with the increased eosinophilia we had observed in mice that underwent neutrophil depletion during allergic sensitization (Figure 3, E and F).

We next studied the effect of neutrophil depletion on the abundance and function of Tregs. Foxp3^–^IL-10^+^ type 1 Tregs made up less than 2% of CD4^+^ T cells, and their numbers were not significantly affected by neutrophil depletion. Foxp3^+^IL-10^–^ Tregs were much more abundant, making up more than 25% of CD4^+^ T cells (Supplemental Figure 6). However, neutrophil depletion did not affect their numbers. We therefore tested whether neutrophil depletion affected the ability of CD25^hi^ Tregs to suppress the proliferation of CFSE-labeled CD4^+^ T effector cells when the 2 cell types were cultured together ex vivo. As expected, CD4^+^ effector cells proliferated ex vivo, as evidenced by dilution of CFSE, and adding CD25^hi^ Tregs to cultures of CD4^+^ effector cells inhibited that proliferation (Figure 5D). However, Tregs from mice that had undergone neutrophil depletion were less effective at suppressing proliferation, suggesting that the increased numbers of eosinophils in neutrophil-depleted mice might stem at least in part from reduced Treg activity.

As group 2 innate lymphoid cells (ILC2s) were not markedly induced during the HDE/OVA model of asthma (Supplemental Figure 7), we hypothesized that neutrophil depletion during sensitization enhanced the pathogenicity of conventional CD4^+^ T cells. To test that, we adoptively transferred naive OVA-specific OT-II T cells into C57BL/6 mice before the animals underwent HDE/OVA sensitization, with or without neutrophil depletion. After 5 daily OVA challenges, total CD4^+^ T cells were isolated from lungs of each group of mice and transferred into separate groups of recipient mice that had already been sensitized twice with HDE/OVA. These animals were then challenged on 3 consecutive days and their lungs subjected to BAL (Figure 5E). Mice given CD4^+^ T cells from donors that received either no antibody or IC antibody during sensitization displayed only modest eosinophilia after 3 challenges. However, mice receiving CD4^+^ T cells from donors undergoing neutrophil depletion during sensitization displayed very robust eosinophilia (Figure 5F). Thus, the presence of neutrophils during allergic sensitization curtails the pathogenicity of developing allergen-specific CD4^+^ T cells, and depleting those neutrophils during sensitization unleashes CD4^+^ T cell pathogenicity.

Although neutrophil depletion during sensitization led to a marked increase in Gata3^+^CD4^+^ T cells after multiple challenges, this increase was not apparent in lung-draining LNs after a single sensitization (Supplemental Figure 8) or after 2 sensitizations (Supplemental Figure 9). Furthermore, neutrophil depletion during sensitization also failed to affect production of cytokines by cells prepared from mediastinal LNs (mLNs) 4 days postsensitization (Supplemental Figure 10). These findings are consistent with our earlier finding that the impact of depleting neutrophils during LPS/OVA-mediated allergic sensitization is significant after multiple daily challenges, but not after sensitization only, or even after a single OVA challenge. Together, these data suggest that depletion of neutrophils during sensitization has a pronounced, but delayed, impact on the phenotype and function of allergen-specific CD4^+^ T cells.

### Neutrophil recruitment to the airway promotes production of TGF-β but not other regulatory cytokines.

**Figure 6:**
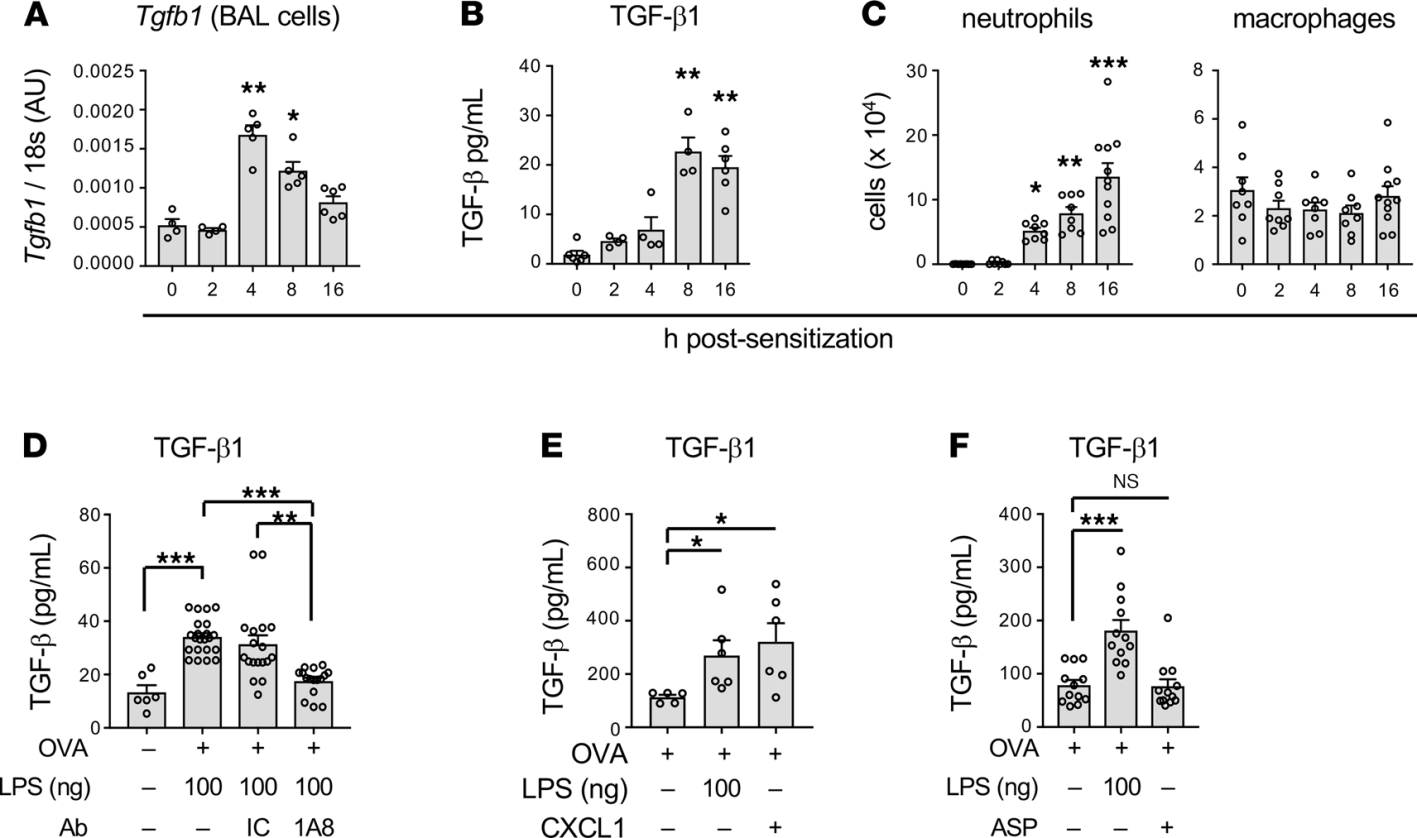
Role of TGF-β in neutrophil-mediated immunosuppression of responses to allergen challenge. (**A**) Relative amounts of *Tgfb1* mRNA in BAL fluid cells at the indicated times after LPS/OVA. Values shown are in arbitrary units (AU), after normalization to 18s mRNA (*n* = 4–6 mice per group). (**B**) Concentrations of TGF-β in BAL fluid at the indicated times after LPS/OVA treatment (*n* = 4–6 mice per group). (**C**) Cell numbers for neutrophils and macrophages at the indicated times post-LPS/OVA instillation (*n* = 8–11 mice per group). (**A**–**C**) Values shown represent mean ± SEM from 1 of 2 experiments yielding similar results. *P < 0.05, **P < 0.01, ***P < 0.001; untreated vs. sensitized mice; Kruskal-Wallis 1-way ANOVA with Dunn’s multiple-comparison test. (**D**) Effect of neutrophil depletion (1A8 administration) on TGF-β concentrations in BAL fluid (*n* = 4–18 mice per group). Values shown represent mean ± SEM from the combined data of 2 experiments yielding similar results. (**E**) Effect of CXCL1 instillation on TGF-β production (*n* = 6 mice/group). Values shown represent mean ± SEM. (*n* = 6 mice per group.) (**F**) Comparison of TGF-β in the airway following either LPS or ASP instillation (*n* = 12 mice/group). Shown are combined data from 2 experiments. (**D**–**F**) *P < 0.05, **P < 0.01, ***P < 0.001; Kruskal-Wallis 1-way ANOVA with Dunn’s multiple-comparison test (**D**) or 1-way ANOVA with Holm-Šídák multiple-comparison test (**E** and **F**).

As neutrophil depletion during the sensitization phase increased allergen-specific CD4^+^ T cell pathogenicity, we reasoned that neutrophils might affect the amounts of 1 or more cytokines produced during LPS-mediated allergic sensitization. IL-10 is one of the best described regulatory cytokines, but neither *Il10* mRNA nor IL-10 protein levels were significantly changed following administration of LPS/OVA (Supplemental Figure 11). Similarly, another regulatory cytokine, IL-27, also remained unchanged (Supplemental Figure 11). However, LPS/OVA treatment did lead to marked increases in *Tgfb1* RNA (Figure 6A), which encodes TGF-β, a well-described regulatory cytokine with many activities, including the ability to promote the differentiation of Tregs. *Tgfb1* expression reached a peak at around 4 hours after LPS/OVA sensitization and began to decline thereafter. TGF-β protein also increased postsensitization, and as might be anticipated, was delayed compared with *Tgfb1* RNA (Figure 6B). These increases occurred in parallel with accumulating neutrophils in the airway (Figure 6C).

The temporal association of the accumulating neutrophils with increasing TGF-β levels prompted us to test whether these 2 observations were causally related. Administration of the neutrophil-depleting anti-Ly6G antibody markedly reduced airway levels of TGF-β compared with animals that received no antibody or the IC antibody (Figure 6D), thus confirming that neutrophils are required for the increased production of TGF-β during LPS-mediated allergic sensitization. By contrast, neutrophil depletion had little effect on production of the proinflammatory cytokines IL-1α, IL-1β, TNF-α, or IL-6 (Supplemental Figure 12). To determine whether neutrophil accumulation in the airway is also sufficient for TGF-β production, we returned to the model in which we elicited airway neutrophilia by instillation of the neutrophil-attracting chemokine, CXCL1. This chemokine, whose inhalation during allergic sensitization increased immunoregulatory responses to subsequent allergen challenge (Figure 4E), also promoted the release of TGF-β into the airway (Figure 6E). Indeed, CXCL1 was as effective in this regard as 100 ng LPS. Conversely, ASP/OVA-mediated allergic sensitization, which does not trigger acute neutrophilia or lead to immunosuppression of established inflammation (Figure 1, B and C), failed to elicit TGF-β production (Figure 6F). Together, these data demonstrate that infiltrating neutrophils are both necessary and sufficient to induce TGF-β production and suggest that this neutrophil/TGF-β axis drives immunosuppressive responses to multiple allergen challenges.

### TGF-β promotes regulatory responses to multiple allergen challenges.

**Figure 7:**
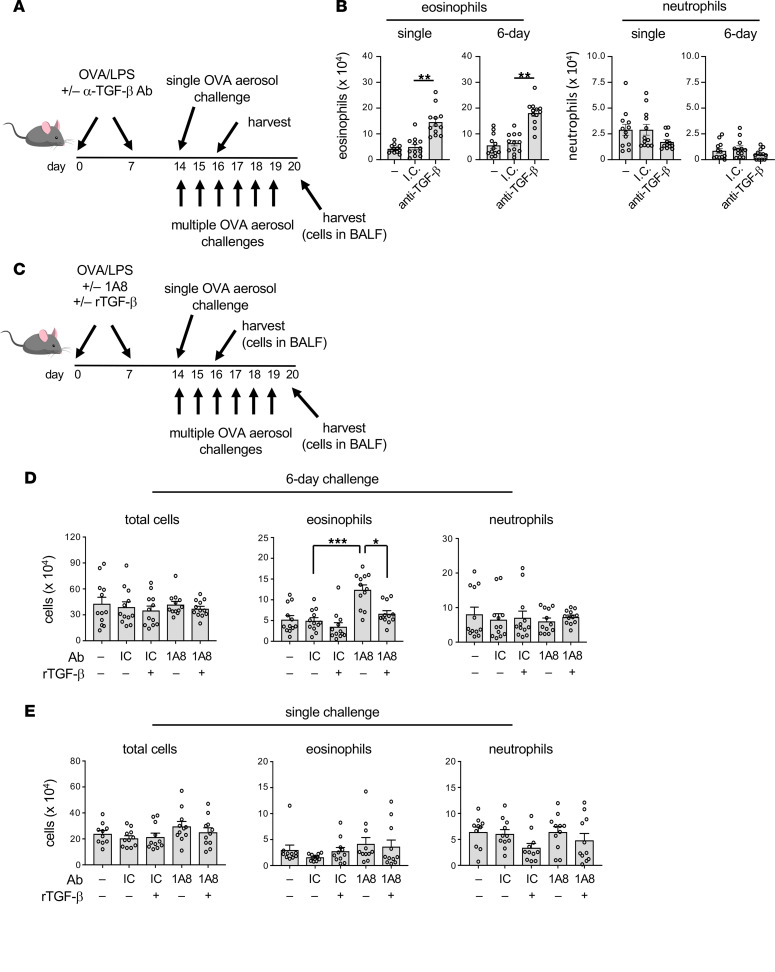
TGF-β blockade during sensitization enhances subsequent responses to allergen challenge. (**A** and **B**) Effect of TGF-β blockade during sensitization on responses to subsequent OVA challenge. (**A**) Timeline for blockade of TGF-β during allergic sensitization, followed by OVA challenge on a single occasion (top) or multiple occasions (bottom). (**B**) Mean cell numbers ± SEM for eosinophils and neutrophils in BAL fluid of mice that received anti–TGF-β Ab or isotype control (IC) Ab prior to sensitization and were challenged once or on multiple occasions. Data shown are from 1 of 2 experiments yielding similar results (*n* = 12 mice/group). **P < 0.01; Kruskal-Wallis 1-way ANOVA with Dunn’s multiple-comparison test. (**C**–**E**) Exogenous rmTGF-β reverses the ability of neutrophil blockade to enhance allergic responses. (**C**) Timeline for allergic sensitization to LPS/OVA, neutrophil depletion, inhalation of exogenous rmTGF-β, followed by a single OVA challenge (top) or multiple OVA challenges (bottom). (**D** and **E**) Effect of rmTGF-β administration on allergic responses of mice undergoing neutrophil depletion. Shown are cell numbers for the indicated cell types in BAL fluid of mice challenged on 6 consecutive days (**D**) or on a single occasion (**E**). Values shown represent mean ± SEM, and data are combined from 2 experiments (*n* = 12 mice/group). *P < 0.05, ***P < 0.001; (**A**) Kruskal-Wallis 1-way ANOVA with Dunn’s multiple-comparison test or (**B** and **C**) 1-way ANOVA with Holm-Šídák multiple-comparison test.

We next tested whether the TGF-β associated with neutrophil infiltration is required for the delayed regulatory responses to multiple allergen challenges. To this end, we blocked TGF-β with a neutralizing antibody (clone 1D11) prior to LPS/OVA-mediated allergic sensitization, then exposed the mice to a single OVA challenge or to 6 daily challenges (Figure 7A). Compared with mice that had received the IC antibody, animals treated with the anti–TGF-β antibody prior to sensitization had much greater eosinophilia after a single OVA challenge and after multiple challenges (Figure 7B). Thus, TGF-β produced during LPS-mediated allergic sensitization through the airway is required for immunoregulatory responses to allergen challenge.

Finally, to confirm the importance of TGF-β in neutrophil-mediated immunosuppression, we tested whether the increased inflammatory responses to multiple daily allergen challenges seen in mice undergoing neutrophil depletion during allergic sensitization could be reversed by adding back recombinant mouse (rm) TGF-β (Figure 7C). As seen in prior experiments, neutrophil depletion during allergic sensitization again increased allergic responses to multiple subsequent allergen challenges; however, the addition of rmTGF-β during sensitization reversed this effect and restored immunosuppression (Figure 7D). Once again, multiple daily OVA challenges were required for this effect, as the addition of rmTGF-β to neutrophil-depleted mice did not significantly affect responses to a single OVA challenge (Figure 7E).

## Discussion

Airway neutrophils can affect multiple features of asthma, including mucus hypersecretion (18–20) and AHR (21). Most of the known functions of neutrophils are associated with exacerbations of asthma, as opposed to a role for these cells in shaping the initiation of immune responses (22). Nonetheless, recent findings suggest that neutrophils that are recruited to the airways during allergic sensitization may drive regulatory activities that function to suppress disease progression. For example, we previously reported that the nature and longevity of immune responses to inhaled OVA are remarkably sensitive to the amounts of LPS used as the adjuvant for allergen sensitization, with higher doses promoting both Th17 responses and shorter lived eosinophilic inflammation, compared with lower doses (11). This finding is consistent with several epidemiologic reports showing that asthma prevalence is inversely associated with household levels of endotoxin (23–26). Our current results suggest that the protective features of endotoxin might stem, at least in part, from its ability to recruit neutrophils to the airway during LPS-mediated allergic sensitization. This recruitment, which does not occur during protease-mediated sensitization, results in increased airway levels of TGF-β that in turn suppress the pathogenicity of allergen-specific Th2 cells. Thus, blockade of neutrophil recruitment during sensitization unleashes the pathogenic potential of allergen-specific CD4^+^ T cells, as evidenced by their heightened ability to drive allergen-specific, eosinophilic inflammation upon their adoptive transfer into naive recipients.

Although the ability of neutrophils to modulate immune responses was first noted in an LPS-mediated model of allergic asthma, we also observed similar effects in 2 different models of asthma in which extracts of common house dust were used as the adjuvant. This suggests that in addition to acting as an adjuvant, endotoxin present in house dust can also elicit neutrophil-dependent regulatory responses that limit the longevity of responses to sustained allergen challenge. Further, these 2 opposing actions of endotoxin might also help explain why some epidemiologic studies find a negative association between household levels of endotoxin and asthma prevalence, whereas other studies find a positive association between these 2 parameters (9, 27).

Our current findings are generally consistent with a recent report by Patel and colleagues showing that neutrophil depletion augments allergic inflammation of the airway in a different model of asthma (28). In that study, BALB/c mice were treated with house dust mite extracts multiple times per week over a 3-week period, and neutrophils were depleted by antibody administration throughout the experiment. By contrast, our current study used C57BL/6 mice, and neutrophils were depleted only during allergic sensitization. Patel and colleagues concluded that in their model, neutrophil depletion enhanced the actions of ILC2s, which were the major cellular source of IL-13 (28). We did not find appreciable numbers of pulmonary ILC2 cells in the models we used, even though these cells could be readily identified in the lung following instillation of ILC2-activating cytokine, IL-33. It is possible that the relative paucity of ILC2s in the mice we studied is a function of the model or mouse strain. ILC2s from BALB/c mice can produce higher amounts of ozone-induced IL-5 and IL-13 than do their C57BL/6 counterparts (29), although this is dependent on the animal model used, as well as the markers used to identify ILC2s (30). However, the specific Th2-associated molecules besides GATA-3 that are affected by neutrophil depletion remain unknown, as the observed increased eosinophilia was not associated with increased amounts of IL-5 or the eosinophil chemoattractants, CCL11 and CCL24 (GS Whitehead et al., unpublished observations). Alternative mechanisms that might have led to the increased eosinophilia include neutrophil-dependent changes in cytokine receptor expression and differential production of other secreted molecules. It is also possible that type 2 cytokines were affected but not at the time points studied here.

Most of our knowledge regarding the roles of TGF-β in Th cell differentiation is based on in vitro studies, which have shown that this cytokine can act alone to promote the development of Tregs or together with the proinflammatory cytokines IL-1 and IL-6 to drive the differentiation of Th17 cells. Interestingly, TGF-β is reported to drive ILC2 expansion (31), which might be anticipated to exacerbate ILC2-dependent allergic airway inflammation. However, neutrophil depletion and consequent reduction in TGF-β in our model was associated with the opposite effect, namely increased eosinophilic inflammation. This further suggests that the ILC2s are not driving the asthma-like phenotype in our models. It is also noteworthy that in our models, neutrophil depletion during sensitization was associated with significantly increased AHR (Figure 3G), whereas this did not occur in the model Patel and colleagues used, again suggesting different mechanisms of actions in the 2 studies.

Despite the well-established ability of TGF-β to promote Treg and Th17 cell development in vitro, the extent to which different levels of naturally occurring TGF-β affect developing allergic responses in the lung is poorly understood. The effect of depleting neutrophils during allergic sensitization on T cell responses to allergen only became apparent after the animals were challenged on multiple occasions. Thus, depleting neutrophils during allergic sensitization did not affect production of type 2 cytokines in lung-draining LNs shortly after sensitization or eosinophilic responses to a single allergen challenge. By contrast, antibody-mediated blockade of TGF-β prior to sensitization was sufficient to increase eosinophilic responses to a subsequent single allergen challenge. One possible explanation for the stronger inflammatory phenotype associated with blockade of TGF-β itself during allergic sensitization is that there are multiple sources of this cytokine, including epithelial cells (28), macrophages, and neutrophils themselves. Thus, depletion of neutrophils does not block all sources of TGF-β. Instead, recruitment of these cells to the airway likely acts to fine-tune levels of TGF-β, which in turn modulates the pathogenic potential of T cells. This regulatory mechanism might have evolved to limit tissue damage associated with type 2 immune responses to relatively harmless antigens, including allergens. Taken together, our current findings add to a growing body of literature that shows indiscriminate blockade of neutrophil function might not always be helpful in the context of allergic asthma (32).

## Methods

### Mice.

C57BL/6J, B6.Cg-Tg(TcraTcrb)425Cbn/J (OT-II), and B6.SJL-*Ptprc^a^ Pepc^b^*/BoyJ (CD45.1) male mice were purchased at 5 to 6 weeks of age from The Jackson Laboratory. The animals were housed in specific pathogen–free conditions and used in experiments at between 6 and 12 weeks of age. All animal studies were reviewed and approved by the Institutional Animal Care and Use Committee (IACUC) at the NIEHS, Research Triangle Park, North Carolina, USA.

### Animal models of asthma.

To sensitize mice, we lightly anesthetized them with isoflurane and gave them 2 oropharyngeal (o.p.) administrations, 1 week apart, of 50 μg LPS-free OVA (Worthington Biomedical) with an adjuvant. The primary adjuvants tested included either 1 or 100 ng LPS from *E*. *coli* 0111:B4 (MilliporeSigma), 20 μg proteases from ASP (MilliporeSigma), or 20 μL of HDEs. All o.p. instillations were in a total volume of 50 μL with sterile PBS as the diluent. Where indicated, neutrophils were depleted 24 and 6 hours prior to sensitization by i.p. injections of 150 μg anti-Ly6G Ab (clone 1A8) (Bio X Cell) or anti–Gr-1 Ab (anti-Ly6C/Ly6G; clone RB6-8C5) (BioLegend). A separate cohort of animals received IC Ab, rat IgG2a (clone 2A3) and rat IgG2b (clone RTK4530). In some experiments, neutrophils were recruited to the airways by o.p. instillation of 0.35 μg mouse CXCL1 (Gemini Bio-products). Where indicated, TGF-β1 was neutralized by 2 i.p. injections of 100 μg anti–TGF-β1 Ab (clone 1D11.16.8) (Bio X Cell), given 24 and 6 hours prior to sensitization, with mouse IgG1 Ab (clone MOPC-21) (Bio X Cell) serving as the IC. In some experiments, 10 ng rmTGF-β (R&D Systems) was administered 4 hours postsensitization by o.p. instillation. For allergen challenges, previously sensitized mice were exposed 7 days after the second sensitization to an aerosol of 1% OVA (MilliporeSigma) in PBS for 1 hour on a single occasion or for 30 minutes on multiple consecutive days. After a single allergen challenge, neutrophils infiltrate the airways very rapidly and peak around 1–2 days postchallenge, whereas eosinophils do not peak until 2–3 days postchallenge. Thus, assessing inflammation 2 days after a single challenge allowed study of neutrophils and eosinophils. By contrast, these cells were present in the airway throughout the multiple challenge protocol, and these mice were harvested 24 hours after the last challenge. Unless otherwise indicated, BAL fluid was collected using 1 mL sterile PBS at the indicated times postsensitization for analysis of cellular inflammation and cytokines, whereas airway inflammation and AHR were assessed 48 hours after a single challenge or 24 hours after the last of several daily OVA challenges.

### Treg function experiments.

CD4^+^CD25^+^ Tregs were sorted from the lungs of C57BL/6 donor mice using a MojoSort Treg kit (BioLegend) and suspended in suspended in complete RPMI (cRPMI) media containing RPMI 1640 (Thermo Fisher Scientific), 10% fetal bovine serum, 0.1% 2-mercaptoethanol, 10 mM HEPES, and 100 U/mL penicillin/streptomycin. For responder cells, naive CD4^+^ T cells were sorted from LNs and spleens of CD45.1 mice using a MojoSort naive CD4 T cell kit (BioLegend), labeled with 3 μM CFSE, and resuspended at 2 × 10^6^/mL. To prepare APCs, CD90.2^–^ cells were isolated from spleens of C57BL/6 mice using a MojoSort CD90.2^+^ cell depletion kit (BioLegend), irradiated with 30 Gy γ-ray, and resuspended at 8 × 10^6^/mL. These 3 cell types (1 × 10^5^ naive CD4^+^ T cells, 5 × 10^4^ Tregs, and 4 × 10^5^ APCs) were cultured together in a total of 200 μL cRPMI-10 containing 125 ng/mL anti–mouse CD3e mAbs (clone 145-2C11, BD Pharmingen) in 96-well, round-bottom plates (with low-evaporation lid, BD Biosciences). Three days later, proliferation of CD45.1^+^ responder CD4^+^ T cells was estimated by measuring the reduction of CFSE signals in flow cytometry on an LSRFortessa flow cytometer (BD Biosciences). Proliferative indices for CD4^+^ T cells from each experimental condition were generated by summing the percentage of cells corresponding to each division multiplied by that division number, thus, (% of undivided cells × 0) + (% of cells that underwent 1 division × 1) + (% of cells that underwent 2 divisions × 2), and so forth up to 6 divisions.

### Adoptive transfer experiments.

OVA-specific CD4^+^ T cells were prepared from spleens and LNs of OT-II mice or CD45.1 X OT-II mice, enriched using a histopaque gradient as previously described (33), and washed 3 times with sterile PBS. A total of 10^7^ cells were transferred into C57BL/6J (CD45.2) cells by retroorbital vein injection. In some experiments, mice that had received OT-II cells prior to sensitization (primary recipients) served as donors of pulmonary CD4^+^ T cells that were transferred into secondary recipient mice. To do this, lungs of primary recipients were enzymatically digested as previously described (21), then pressed through a 70 μm strainer, and the leukocytes were enriched using a histopaque gradient. CD4^+^ T cells were then purified from leukocytes by negative selection using biotin-labeled antibodies against MHC2 (1-A; clone Af6-120.1, BioLegend), CD11c (clone HL3, BD Pharmingen), CD11b (clone M1/70, BD Pharmingen), CD44 (clone 1M7, BD Pharmingen), CD45R (eBioscience, clone RA3-6B2), and CD8a (clone 53-6.7, BD Pharmingen); streptavidin beads (Miltenyi Biotec); and an autoMACS Pro separator (Miltenyi Biotec). These purified CD4^+^ T cells (1.5 × 10^6^) were adoptively transferred by retroorbital vein injections into secondary recipient mice that had been previously sensitized to OVA. In other experiments, naive CD4^+^ T cells were isolated from spleens and LNs of OT-II mice, then differentiated in vitro to Th2 cells as previously described (7), and 10^6^ cells were transferred into mice that had been previously instilled with LPS.

### T cell development in lung-draining mLNs.

Enriched CD4^+^ T cells prepared from spleens and LNs of OT-II transgenic mice were transferred (10^7^ cells) by retroorbital vein injection into recipient mice. These animals were then sensitized by o.p. instillation of OVA with a test adjuvant. Four days later, mLNs were excised, minced, and pressed through a 70 μm strainer, and 1 × 10^6^ cells were cultured in 200 μL cRPMI media containing 10 μg/mL OVA. Culture supernatants were analyzed for cytokines.

### Analysis of cytokines in BAL fluid and supernatant of cultured regional LNs.

Concentrations of TGF-β protein in whole lung lavage fluid were determined using commercial ELISA kits specific for activated mouse TGF-β according to the instructions of the manufacturer (R&D Systems). Concentrations of IL-4, IL-5, IL-10, and IL-17 in BAL fluid or the supernatant of cultured regional LNs were measured as described previously (7), using a multiplexed fluorescent bead-based immunoassay (Bio-Rad Laboratories).

### Analysis of RNA.

Following whole lung lavage, the cells in BAL fluid were collected by centrifugation (450*g*, 5 minutes, 4°C), then flash-frozen in liquid N_2_, and the RNA was isolated using a Qiagen RNeasy Mini Kit. Quantitative PCR was performed to assess *Tgfb1* RNA per the instructions of the manufacturer (Applied Biosystems), using a primer set from PrimerBank (ID 6755774c2), forward primer 5′-GAGCCCGAAGCGGACTACTA-3′ and reverse primer 5′-TGGTTTTCTCATAGATGGCGTTG-3′. Values were normalized to expression of the housekeeping gene *18s*, forward primer 5′-CGGCTACCACATCCAAGGAA-3′ and reverse primer 5′-GCTGGAATTACCGCGGCT-3′.

### Flow cytometry.

Lungs were excised, minced, and enzymatically digested. Lymphocytes were enriched using a histopaque gradient, diluted to 2 × 10^6^/100 μL, and incubated with a nonspecific binding blocking reagent cocktail of anti-mouse CD16/CD32 (2.4G2), normal mouse, and normal rat serum (Jackson ImmunoResearch) for 10 minutes. Cells were then stained with fluorochrome-labeled antibodies against mouse CD4 (BD Biosciences catalog 740208; clone RM4-5) and CD25 (BD Biosciences catalog 551071; clone PC61), then fixed prior to intracellular staining for the mouse transcription factors Gata3 (BioLegend catalog 653810; clone 16E10A23), T-bet (BioLegend catalog 644817; clone 4B10), RORγt (BD Biosciences catalog 562684; clone Q31-378), and Foxp3 (Invitrogen 48-5773-82; clone FJK-16s). In some experiments, donor CD45.1 cells and recipient CD45.2 cells were identified by staining with antibodies against mouse CD45.1 (eBioscience catalog 12-0453-81; clone A20) and CD45.2 (BD Pharmingen catalog 553772; clone 104). Cells were evaluated using an LSR II flow cytometer (BD Biosciences), and data were analyzed using FlowJo software, version 9.6 (Tree Star).

### AHR.

Evaluations of AHR were performed as previously described (21), using the FlexiVent mechanical ventilator system (Scireq). A single-compartment model of the lung was used to assess total respiratory system resistance after delivery of aerosolized methacholine using an ultrasonic nebulizer (Scireq). Data are reported as peak resistance values.

### Statistics.

Most statistical calculations were performed using GraphPad Prism 7 (GraphPad Software, Inc.). Data are shown as mean ± SEM. Differences between groups were identified by Kruskal-Wallis 1-way ANOVA with Dunn’s multiple-comparison test, 1-way ANOVA with Dunnett’s or Holm-Šídák multiple-comparison tests, or 2-way ANOVA with Tukey’s multiple-comparison test. Individual comparisons between groups were confirmed by 2-tailed Student’s *t* test or the Mann-Whitney *U* test, and a *P* value of less than 0.05 was considered statistically significant.

### Study approval.

All animal experiments were conducted in accordance with and with the approval of the IACUC at NIEHS/NIH.

## Author contributions

GSW, SYT, CGB, HN, and DJR designed and performed experiments with animals and analyzed data. KN and SYT generated Th2 cells for adoptive transfer. GSW and DNC wrote the manuscript, and all authors made editorial suggestions and approved the final version.

## Supplementary Material

Supplemental data

## Version 1 02/22/2022

Electronic publication
