# Supplementary material for: A neutrophil/TGF-β axis limits the pathogenicity of allergen-specific CD4+ T cells
Source: JCI Insight. 2022 Feb 22;7(4):e150251. doi: 10.1172/jci.insight.150251 (PMC8876454; doi:10.1172/jci.insight.150251)
Supplement: Supplemental data [file jciinsight-7-150251-s044.pdf]

Supplemental Figure S1

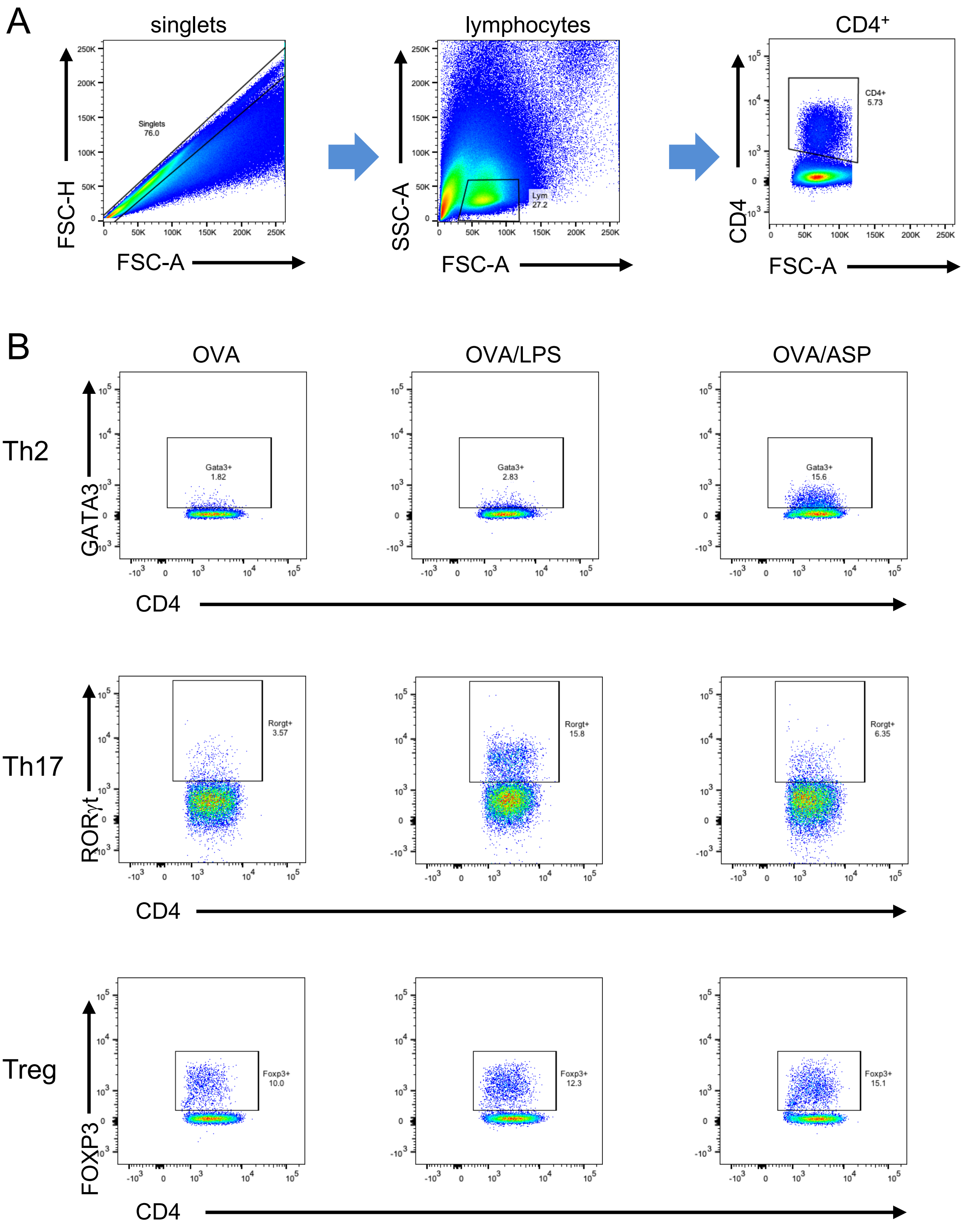

Supplemental Figure S1. Gating strategy and intracellular staining of CD4^{+} T cells. (**A**) Gating strategy for single CD4^{+} T lymphocytes (additional live cell gate not shown). (**B**) Cytograms showing intracellular staining for the indicated master transcription factors in CD4^{+} T cells from lungs of mice sensitized using OVA alone, OVA/LPS or OVA/ASP, and challenged with OVA.

Supplemental Figure S2

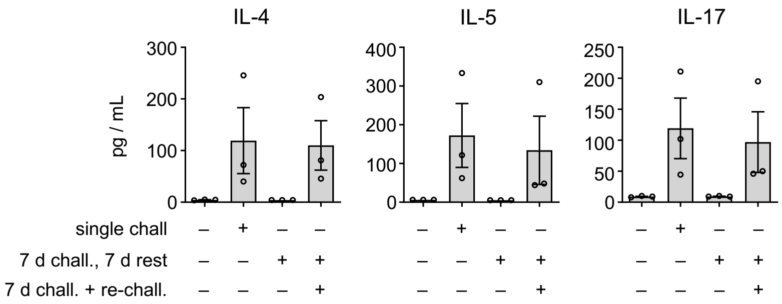

Supplemental Figure S2. Effect of rest following multiple OVA challenges on cytokine response to re-challenge. Shown are mean values ± SEM for concentrations of the indicated cytokines in BALF of mice that were sensitized with LPS/OVA, then challenged (chall), rested, and re-challenged (re-chall) as indicated in **Figure 2A**.

Supplemental Figure S3

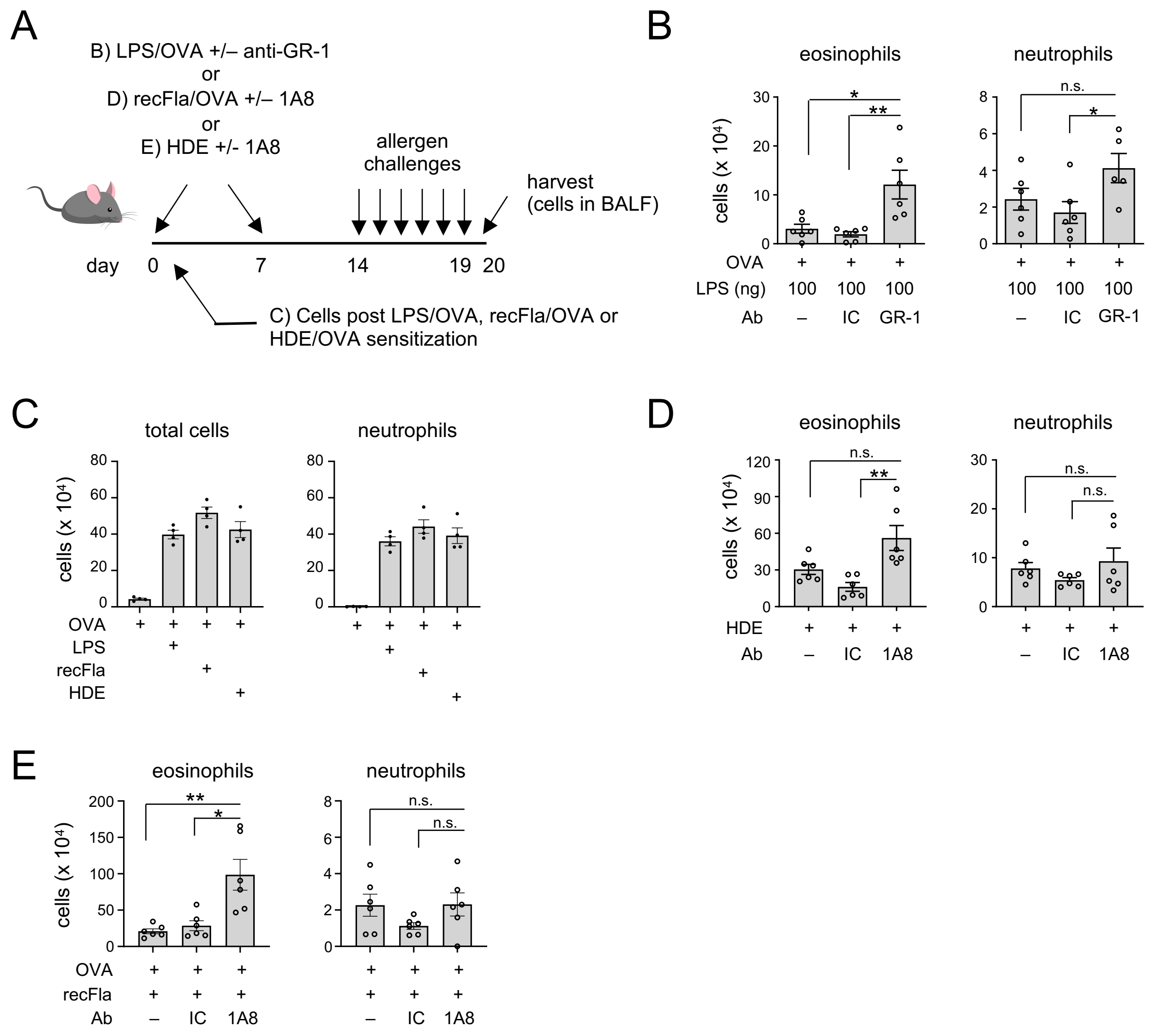

Supplemental Figure S3. Effect of neutrophil depletion during sensitization on subsequent responses to multiple allergen challenges. (**A**) Timeline for neutrophil depletion, allergic sensitization and multiple allergen challenges. (**B**) Mean cell numbers ± SEM for eosinophils and neutrophils in BALF of mice treated with GR-1 or isotype control (IC) Ab during LPS/OVA sensitization and then daily challenges to OVA. (**C**) Neutrophils in BALF 16 hrs after sensitization to OVA with the indicated adjuvant. (**D, E**) Mean cell numbers ± SEM for eosinophils and neutrophils in BALF of mice treated with anti-Ly6G (clone 1A8) or isotype control (IC) Ab during HDE sensitization and challenge (**D**) or recFla/OVA sensitization and OVA challenge (**E**). (**B, D, and E**) *N* = 5-6 mice/group and are from single experiments. *P < 0.05, **P , 0.01. One-way ANOVA with Sidak's multiple comparison test. (**C**) *N* = 4 mice/group and is from a single experiment.

Supplemental Figure S4

A

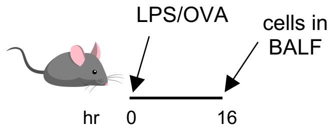

B

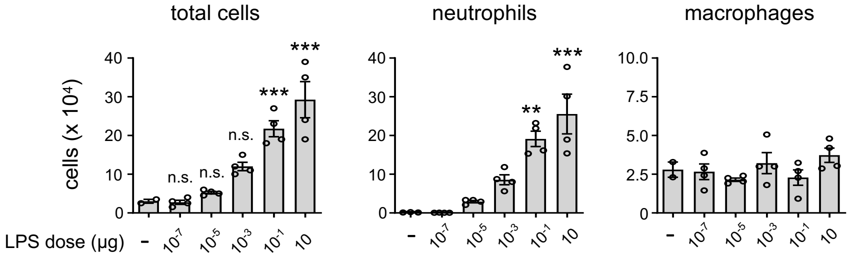

Supplemental Figure S4. **Dose-response for LPS-mediated neutrophil recruitment to the airway**. **(A)** Timeline for instillation of OVA with the indicated amounts of LPS plus OVA, followed by harvest 16 h later. **(B)** Mean cell numbers ± SEM for the indicated leukocytes in BALF of mice after LPS/OVA instillation, as depicted in **A**). (*n* = 4 mice/group). Results are from a single experiment, representative of two. Statistical comparisons are between the different LPS-treated groups and mice treated with OVA only, determined using Kruskal-Wallis one-way ANOVA with Dunn's multiple comparison test. Not significant (n.s.). *P < 0.05, **P < 0.01, ***P < 0.00.

Supplemental Figure S5

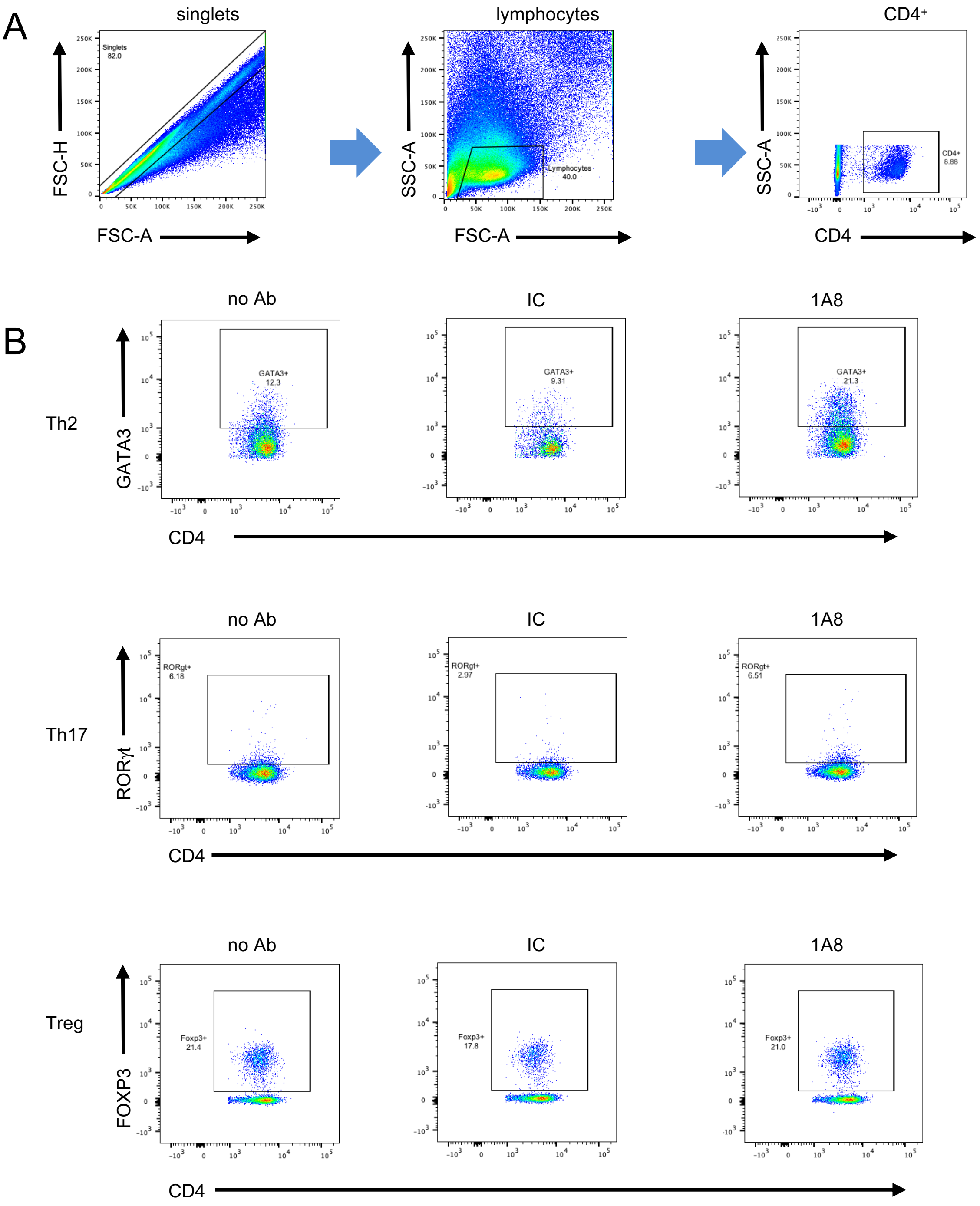

Supplemental Figure S5. Gating strategy and intracellular staining of master transcription factors in CD4⁺ T cells. (**A**) Gating strategy for single, live CD4⁺ lymphocytes. (**B**) Intracellular staining for the transcription factors, GATA3, RORγt, and FOXP3 in CD4⁺ T cells isolated from lungs of mice treated with no Ab, IC, or 1A8 prior to allergic sensitization.

Supplemental Figure S6

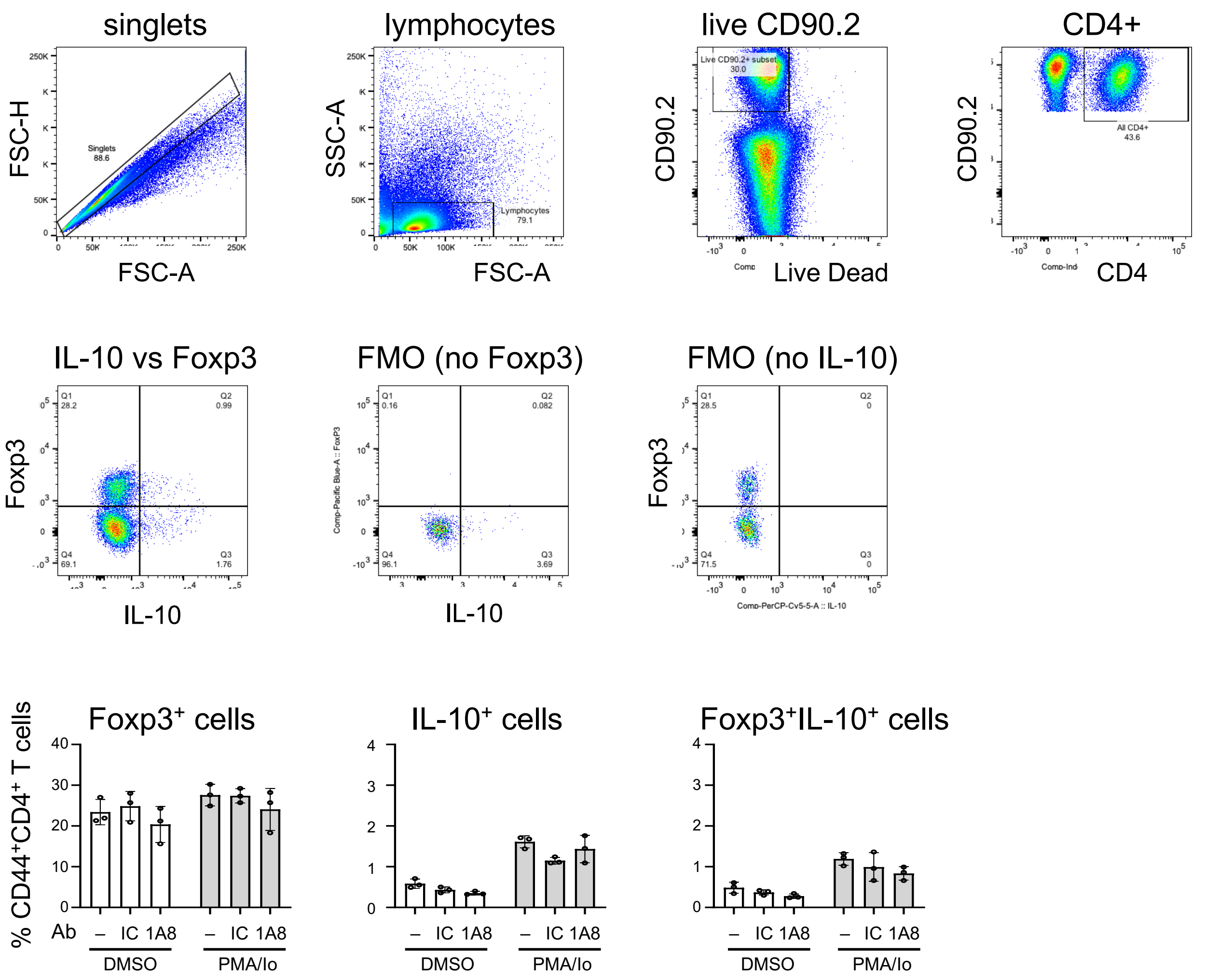

Supplemental Figure S6. Gating strategy and intracellular staining of intracellular Foxp3 and IL-10 in CD4⁺ T cells. Gating strategy for single, live CD90.2⁺ CD4⁺ lymphocytes expressing Foxp3, IL-10, or both cytokines (top) and effect of neutrophil depletion (1A8 antibody) on intracellular staining of cells treated with PMA and Ionomycin (Io), or the vehicle, DMSO.

Supplemental Figure S7

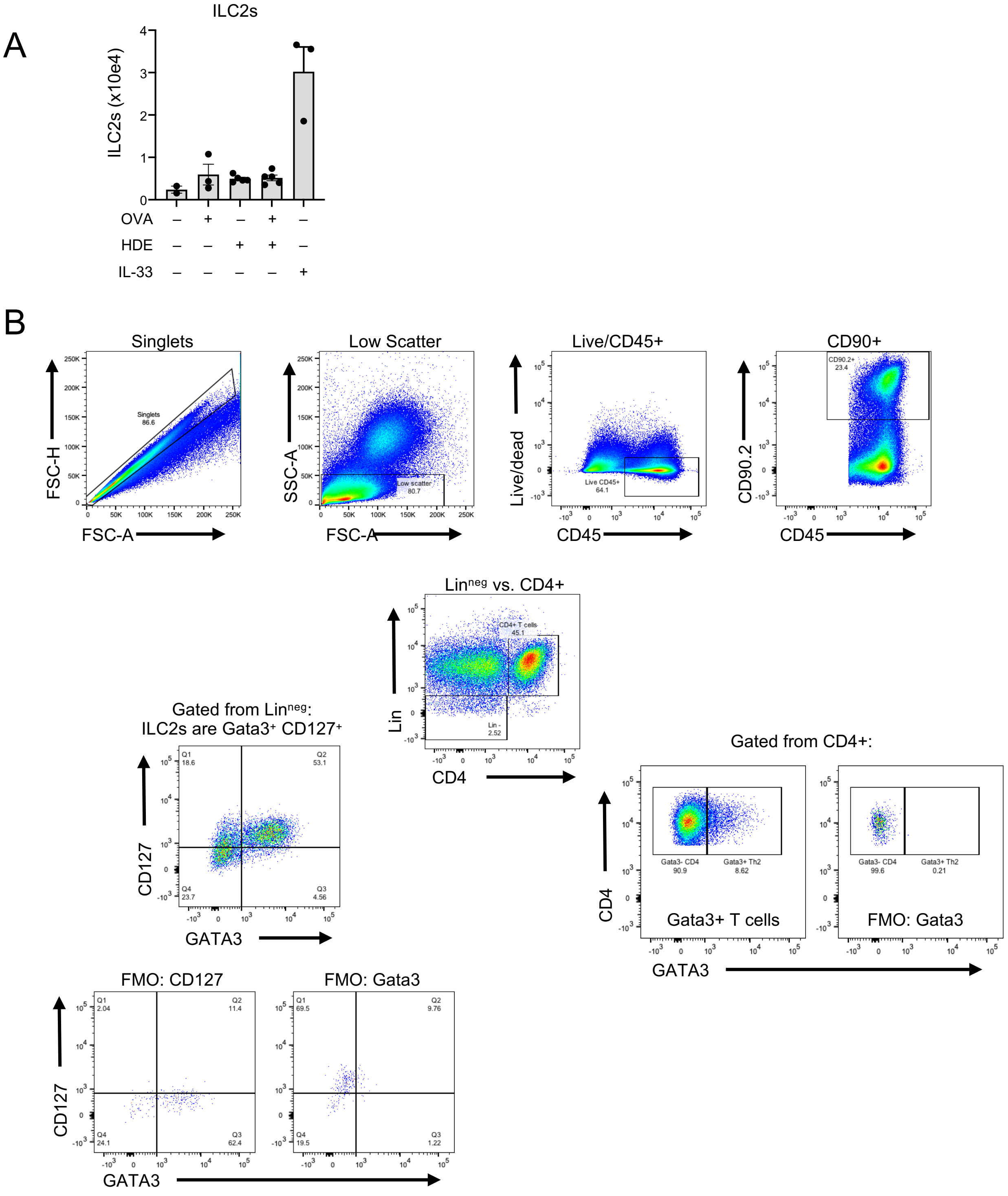

Supplemental Figure S7. Analysis of ILC2 cells in the HDE/OVA model of asthma. (**A**) Numbers of ILC2 cells in lungs of mice treated with OVA, HDE, or with the positive control cytokine, IL-33. (**B**) Gating strategy for single, live, CD90^{+} lymphocytes that are either Lin^{neg} or positive for CD4. CD127 and GATA3 staining for Lin^{neg} cells is also shown, as well as GATA3 staining for CD4^{+} T cells, including fluorescence minus one (FMO) controls.

Supplemental Figure S8

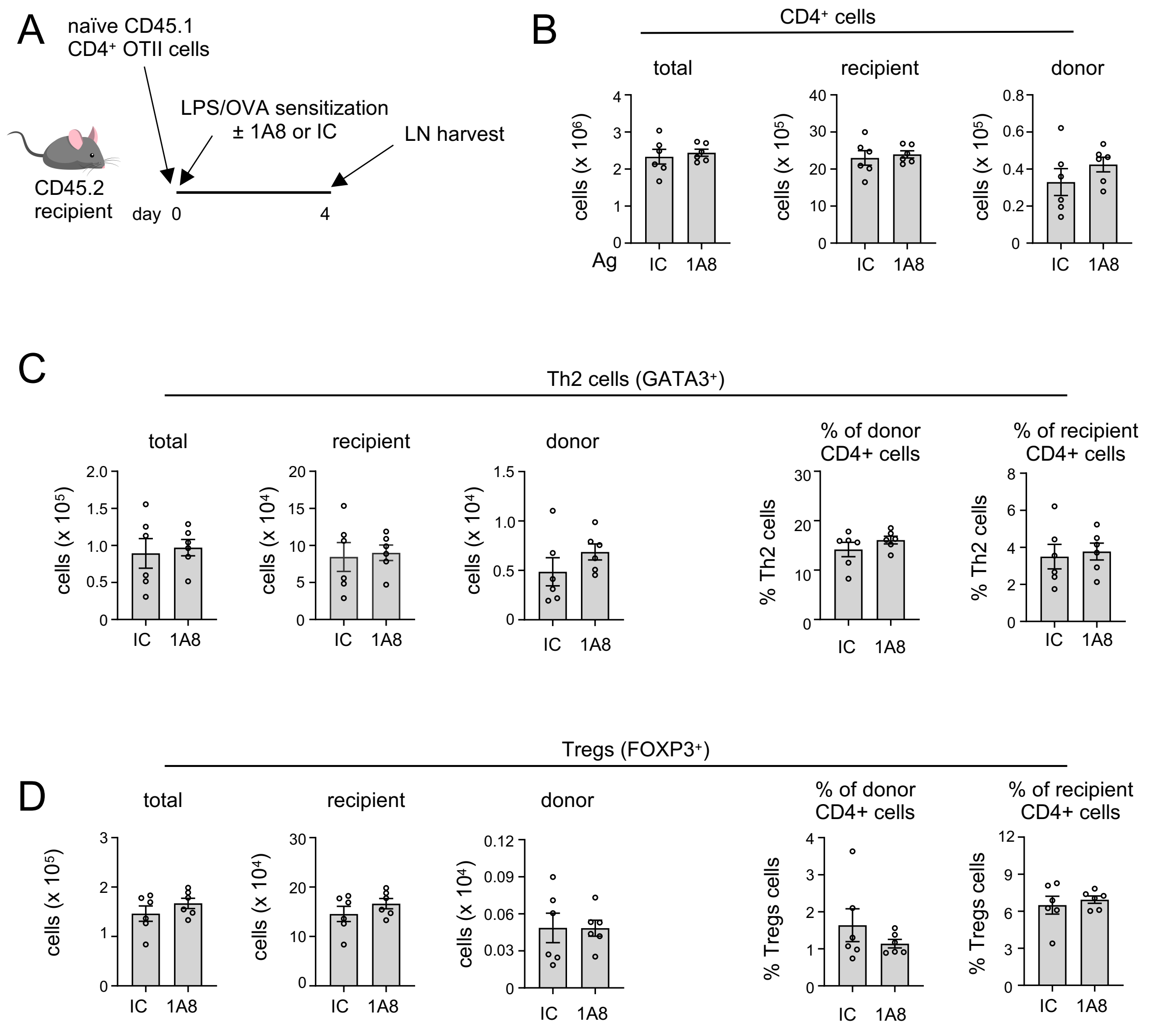

Supplemental Figure S8. Effect of neutrophil depletion during sensitization on CD4⁺ T cell development in regional LNs. (A) Timeline for (CD45.1) OT-II cell adoptive transfer, neutrophil depletion, allergic sensitization and regional LN harvest. (**B**) Total numbers of CD4⁺ T cells derived from adoptively transferred donor cells and recipient mice. (**C,D**) Numbers and percentages of Th2 cells (**C**) and Tregs (**D**) derived from donor cells (CD45.1) and recipient mice (CD45.2). (N = 6 mice/group). *P < 0.05, Kruskal-Wallis one-way ANOVA with Dunn's multiple comparison test. Statistical comparisons are between the different LPS-treated groups and PBS-treated mice.

Supplemental Figure S9

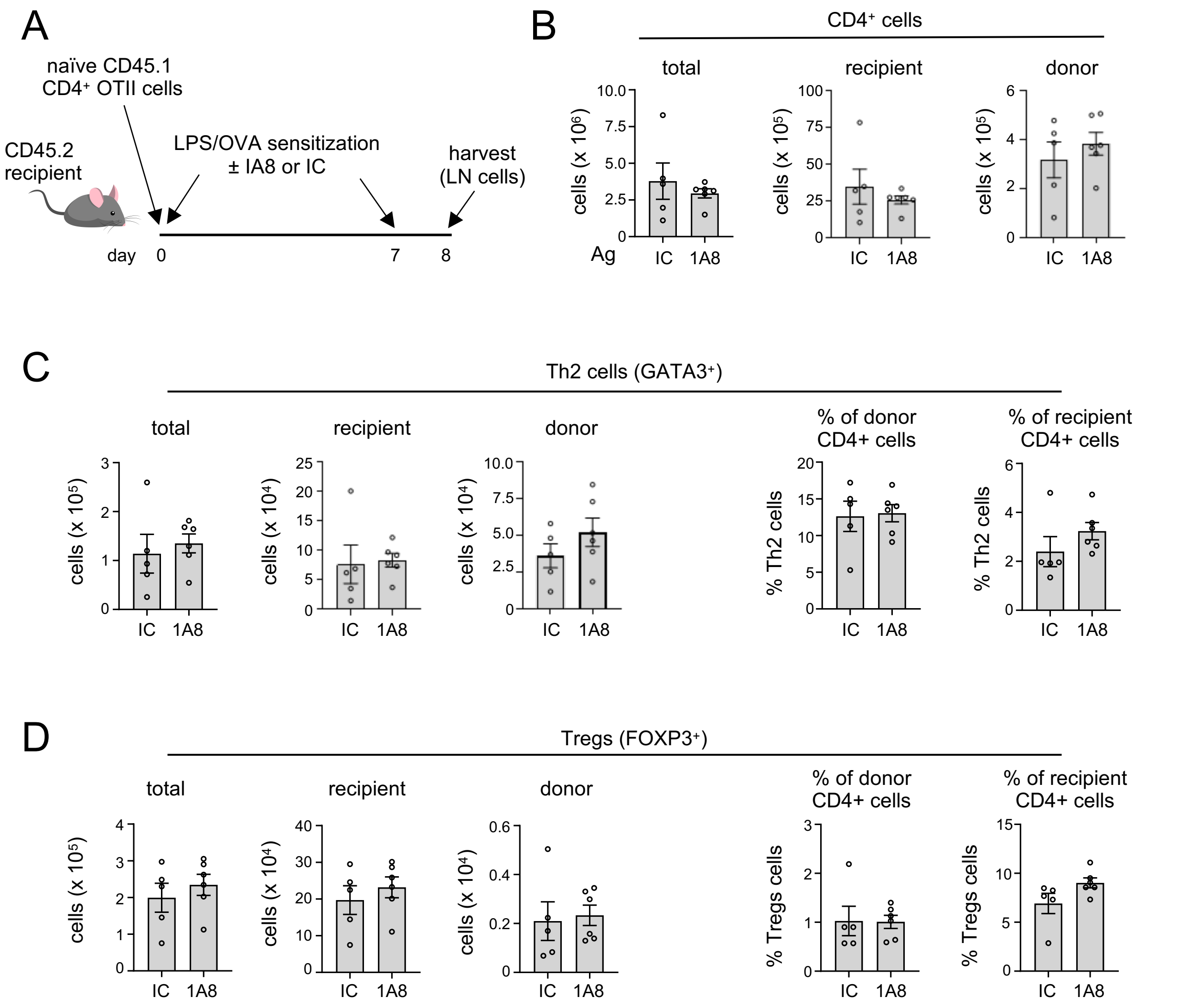

Supplemental Figure S9. Effect of neutrophil depletion during two sensitizations on CD4+ T cell development in regional LNs. (A) Timeline for OT-II cell adoptive transfer, neutrophil depletion, allergic sensitizations and regional LN harvest. (**B**) Total numbers of CD4+ T cells derived from adoptively transferred donor cells and recipient mice. (**C,D**) Numbers and percentages of Th2 cells (**C**) and Tregs (**D**) derived from donor cells (CD45.1) and recipient mice (CD45.2). (n = 6 mice/group). *$P < 0.05$, Kruskal-Wallis one-way ANOVA with Dunn's multiple comparison test. Statistical comparisons are between the different LPS-treated groups and PBS-treated mice.

Supplemental Figure S10

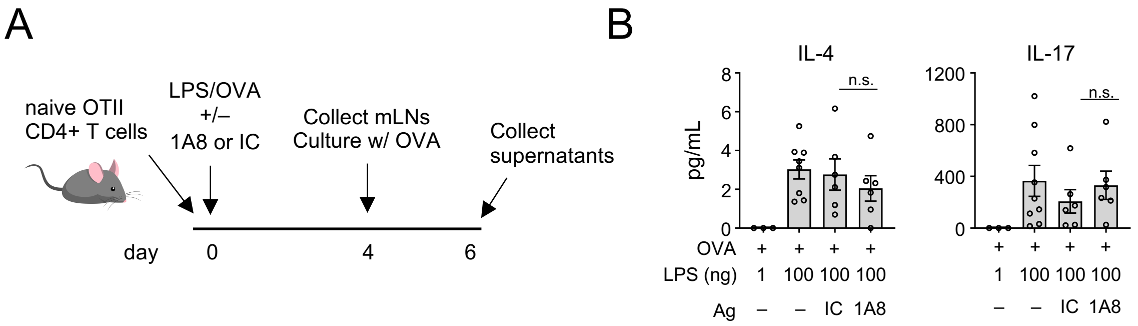

Supplemental Figure S10. Effect of neutrophil depletion during sensitization on cytokine production in regional LNs. (A) Timeline for OT-II cell adoptive transfer, neutrophil depletion, LPS/OVA-mediated allergic sensitization, and regional LN harvest. (**B**) Concentrations of IL-4 and IL-17 in supernatants of LNs harvested from mice treated as indicated and cultured ex vivo. (*N* = 6 mice/group). n.s., non-significant; one-way ANOVA with Sidak's multiple comparison test.

Supplemental Figure S11

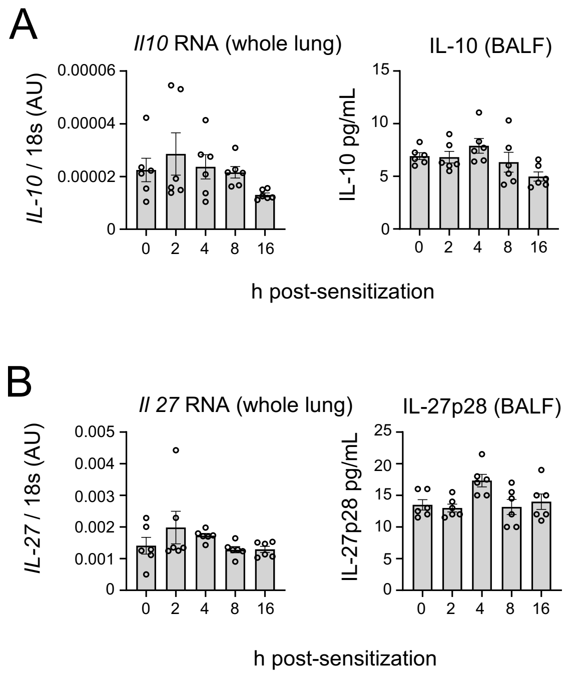

Supplemental Figure S11. RNA expression and protein production of regulatory cytokines following instillation of LPS/OVA. RNA expression values for *Il10* (**A**) and *Il27p2*8 (**B**). Cytokine RNAs were normalized to the housekeeping gene 18S RNA and are presented as arbitrary units (AU).

Supplemental Figure S12

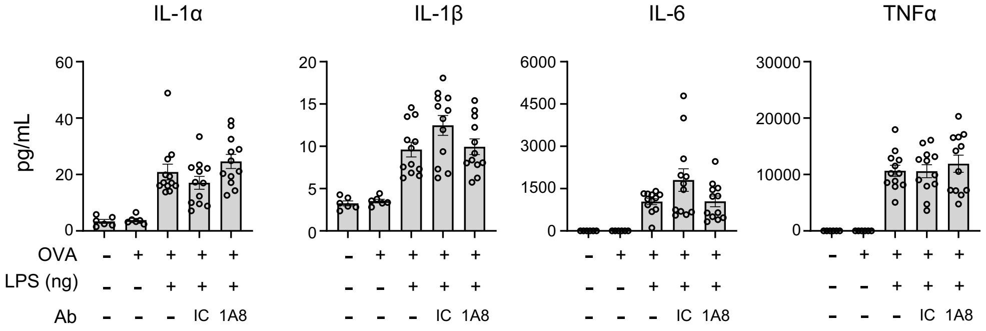

Supplemental Figure S12. Effect of neutrophil depletion during sensitization on production of inflammatory cytokines. Mice were treated with the neutrophil-depleting Ab, 1A8, or with IC, and then sensitized with OVA alone, or LPS/OVA, as indicated. Shown are mean concentrations of the indicated cytokines ± SEM in BALF 4 h post-sensitization. Results are from the combined data of 2 experiments with similar results (*N* = 6 mice for the naïve and OVA alone groups, 12 mice for the LPS/OVA treated groups). Kruskal-Wallis one-way ANOVA with Dunn's multiple comparison test.
